# Sea level fall during glaciation stabilized atmospheric CO_2_ by enhanced volcanic degassing

**DOI:** 10.1038/ncomms15867

**Published:** 2017-07-06

**Authors:** Jörg Hasenclever, Gregor Knorr, Lars H. Rüpke, Peter Köhler, Jason Morgan, Kristin Garofalo, Stephen Barker, Gerrit Lohmann, Ian R. Hall

**Affiliations:** 1 GEOMAR Helmholtz Centre for Ocean Research Kiel, Wischhofstr. 1-3, 24159 Kiel, Germany; 2 University of Bremen, Faculty Geosciences, Klagenfurter Str. 2-4, 28359 Bremen, Germany; 3 Alfred-Wegener-Institut Helmholtz-Zentrum für Polar-und Meeresforschung (AWI), P.O. Box 12 01 61, 27515 Bremerhaven, Germany; 4 School of Earth and Ocean Sciences, Cardiff University, Cardiff CF10 3AT, UK; 5 Department of Earth Sciences, Royal Holloway, University of London, Egham, Surrey TW20 0EX, UK

## Abstract

Paleo-climate records and geodynamic modelling indicate the existence of complex interactions between glacial sea level changes, volcanic degassing and atmospheric CO_2_, which may have modulated the climate system’s descent into the last ice age. Between ∼85 and 70 kyr ago, during an interval of decreasing axial tilt, the orbital component in global temperature records gradually declined, while atmospheric CO_2_, instead of continuing its long-term correlation with Antarctic temperature, remained relatively stable. Here, based on novel global geodynamic models and the joint interpretation of paleo-proxy data as well as biogeochemical simulations, we show that a sea level fall in this interval caused enhanced pressure-release melting in the uppermost mantle, which may have induced a surge in magma and CO_2_ fluxes from mid-ocean ridges and oceanic hotspot volcanoes. Our results reveal a hitherto unrecognized negative feedback between glaciation and atmospheric CO_2_ predominantly controlled by marine volcanism on multi-millennial timescales of ∼5,000–15,000 years.

## Abstract (web summary)

Ice core data show a breakdown in the long-term temperature-atmospheric CO_2_ correlation during interglacial-glacial transitions. Here, via a novel modelling approach, the authors reveal marine volcanism, triggered by a fall in sea level, as a likely mechanism for the observed delayed decline in atmospheric CO_2_.

Earth’s geosphere and climate have recently been shown to closely interact on orbital time scales rather than being independent sub-systems^1^,^2^,^3^,^4^,^5^,^6^,^7^,^8^. During the end of the last ice age, a positive feedback may have existed between deglaciation, terrestrial volcanism and atmospheric CO_2_ (ref. 1). Likewise, crustal production at mid-ocean ridges (MOR) is now thought to be sensitive to glacial sea level changes^1^,^2^,^3^. This is supported by changes in hydrothermal iron flux variability that indicate a relatively swift increase in hydrothermal activity to the most pronounced sea level fall during the last 50 kyr on a suborbital timescale^6^, possibly within a few thousand years^7^. Such interactions may also have played a role for the different trends in temperature and atmospheric CO_2_ (ref. 9) during a phase of pronounced ice sheet growth and associated sea level fall across the Marine Isotope Stage (MIS) 5/4 boundary (Fig. 1) as corroborated by records of enhanced hydrothermal activity across MIS4 in sediment cores at 38°N on the Mid-Atlantic Ridge^10^, the Galapagos Microplate^11^ and the East Pacific Rise^6^. Between ∼85–70 kyr ago Antarctic and Greenland temperatures display a gradual decline (Fig. 1c), superposed by millennial-scale variability. In contrast, atmospheric CO_2_ remains relatively stable before dropping abruptly towards the end of this interval (Fig. 1d). Hence, the close correlation between atmospheric CO_2_ and Antarctic temperature throughout most of the last 800 kyr^12^ transiently breaks down across the MIS 5/4 transition^9^. With respect to the relatively stable CO_2_ levels between ∼85 and 70 kyr ago one could ask in an idealized sense what ‘held-up’ CO_2_ concentrations during the gradual global temperature decline or, alternatively, why CO_2_ dropped so abruptly at the onset of MIS 4. Previous studies have so far focused on finding answers to the latter question in the framework of physical and biogeochemical processes in the coupled ocean–atmosphere system^13^,^14^. However, the carbon cycle sub-system in current climate models does not fully account for variations in volcanic degassing over a glacial cycle. In this context, it is particularly interesting that this period was accompanied by a sea level fall^15^,^16^,^17^ likely in the order of a several tens of metres (up to ∼100 m in extreme scenarios) within a period of ∼5–15 kyr (Fig. 1f and Supplementary Table 1). This points to the possibility that a transient phase of enhanced volcanic CO_2_ degassing in response to the sinking sea level ‘held up’ atmospheric CO_2_ concentrations at a time of global temperature decline and ice sheet growth.

Here we test this hypothesis by using geodynamic models to compute the likely increases in volcanic CO_2_ flux across the MIS 5/4 boundary at 43 mantle plume-related oceanic hotspot volcanoes and along the global MOR system. All geodynamic simulations have been done with the M2TRI- and M3TET-codes (see ref. 18 and Methods section), which solve for two- (2D) and three-dimensional (3D) thermo-mechanical convection, respectively, including fractional wet melting of the Earth’s mantle. Carbon dioxide is implemented as a passive highly incompatible component that preferentially enters the melt phase during partial melting (Methods section). These models predict that a falling sea level causes enhanced pressure-release melting in the oceanic uppermost mantle. In conjunction with the interpretation of proxy data as well as biogeochemical simulations, we show that the enhanced melting induced a surge in magma and CO_2_ fluxes from oceanic hotspot and MOR volcanoes, which provides a negative feedback between glaciation and atmospheric CO_2_ on multi-millennial timescales.

## Results

### Response of oceanic hotspot volcanoes to sea level fall

Oceanic hotspot volcanoes, unlike MOR^1^,^2^,^3^,^5^, have not yet been investigated for their sensitivity to glacial sea level changes, in spite of a comparatively higher CO_2_ concentration in the plume source^19^,^20^. Furthermore, the special geometry of hotspot volcanoes, which often features a subaerial volcano on top of a much wider submerged volcanic edifice and mantle melting region, makes them susceptible to sea level-linked variations in melt production (Figs 2 and 3). A fall in sea level may further decompress the upwelling mantle, which results in enhanced pressure-release melting and the efficient partitioning of highly incompatible CO_2_ into the melt phase within the lower part of the melting zone (Fig. 2a). Mantle plume melting is inherently 3D and controlled by parameters such as strength and chemical composition of the plume, but it is also shaped by the speed and age of the overriding plate. We have designed a global hotspot-melting model by integrating the results of 126 3D numerical experiments that resolve the key aspects of mantle plume melting with data on plume buoyancy fluxes, plume excess temperatures, plate speeds and plate ages for 43 oceanic hotspots (see Supplementary Table 2 and Methods section). This model predicts a global annual oceanic hotspot magma flux of 2.02 km^3^ (Fig. 2), which is in accord with independently derived estimates of plume-related melt production being about 10% of the global MOR melt production of about 21 km^3^ per year^19^,^21^.

**Figure 1:**
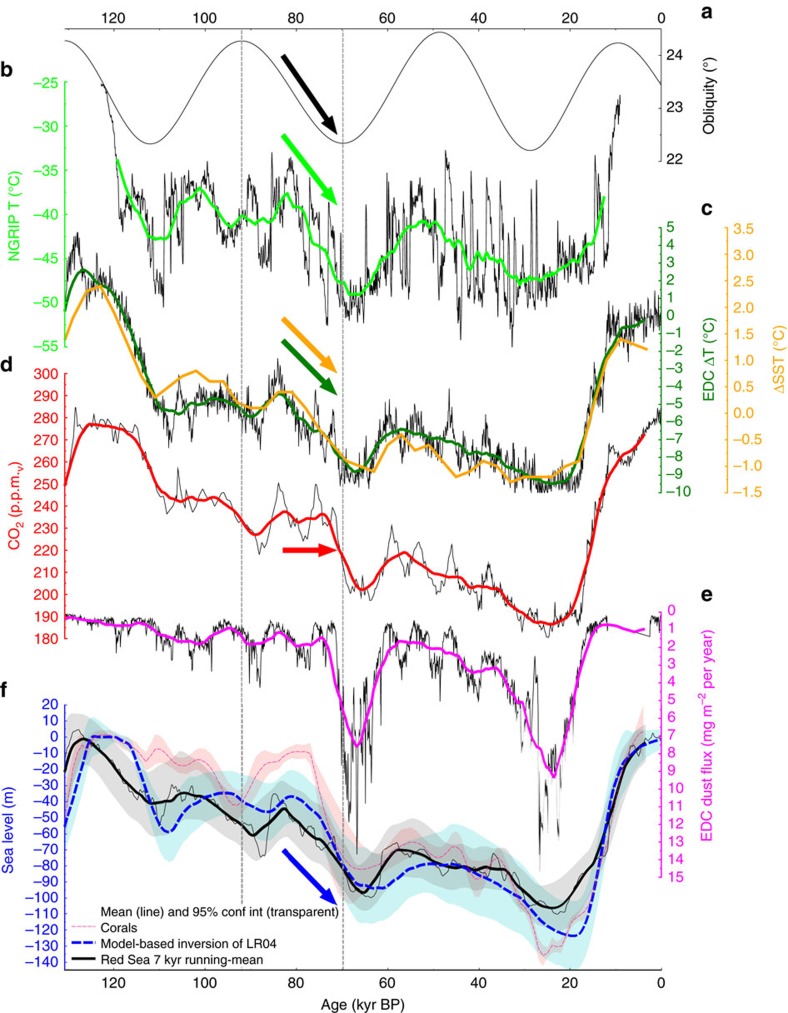
Relevant paleo data during the last glacial cycle. (**a**) Earth’s axial tilt (obliquity)^66^. (**b**) Calculated temperatures from the NGRIP ice core in Greenland on modelled chronology ‘ss09sea09bm’ with 10 years time steps^67^. (**c**) ΔT of EPICA Dome C in Antarctica^68^ on AICC2012 chronology^69^ (black line) and a recent stack of global sea surface temperature (SST) changes^70^ (orange line). (**d**) Atmospheric CO_2_ from the most recent CO_2_ stack on individual age models^39^. (**e**) Dust fluxes to EPICA Dome C^37^ on AICC2012 chronology. (**f**) Estimates of sea level changes. Solid black line with grey band is a Red Sea data-based 95% probability envelope^15^. Dashed blue line with cyan band: ice sheet-based deconvolution of a deep ocean benthic δ^18^O stack with 2 sigma uncertainty range^16^. Light red band: Monte-Carlo-based 95% probability to meet a compilation of U/Th-dated corals^17^ with own calculated mean (dark red broken line). Vertical dashed lines mark the decreasing phase in obliquity around the MIS 5/4 boundary, and coloured arrows highlight where atmospheric CO_2_ is apparently decoupled from long-term trends in temperatures. Antarctic ice core time series are interpolated to time steps of 100 years. The thick coloured lines in all subplots show 7 kyr running means to highlight the long-term orbitally driven changes. The time series based on global stacks of δ^18^O (ΔSST) and ice sheet simulation-based sea level (cyan band in **f**) contain only the orbital-driven signals and are therefore shown as published (without filtering).

To gain a process-based understanding of the impact that a falling sea level has on plume melting and volcanic CO_2_ degassing, we apply a range of different sea level variations (Supplementary Table 1) as derived from the sea level curves shown in Fig. 1f. In the following we focus on the results obtained for a scenario (S2), in which sea level dropped by 60 m over a period of 15,000 years. Linking a sea level forcing to extra decompression melting requires an assessment of the flexural response of the lithosphere as the pressure signal may be damped beneath the subaerial parts of the volcanic edifice. For this purpose, we have performed 3D viscoelastic simulations (see Fig. 3 and Methods scetion) that constrain the damping effect for conical-shaped islands of different sizes. The damping effect scales with island size and we find a maximum local damping of 25% for the largest considered ocean islands (for example, Big Island of the Hawaiian archipelago). In the calculations of increased pressure release melting during a falling sea level, the pressure signal below the respective island size is used for each considered mantle plume (Supplementary Table 2). With this methodology, we find an average transient increase in global plume-related magma flux of 12% corresponding to 0.24 km^3^ per year. It should be noted that Iceland was excluded from this analysis as its magmatic response may have been controlled by ice sheet loading rather than by a falling sea level.

### Response of mid-ocean ridges to sea level fall

MOR melting has recently been shown to be sensitive to glacial sea level changes showing spreading rate dependent variations of 15–100% with respect to the average long-term magma flux^2^,^3^. To obtain a quantitative assessment of the relative importance of these two oceanic subsystems we here additionally present a global MOR melting model. For this purpose, we have integrated a large number of 2D geodynamic simulations (Fig. 4a), assuming full spreading rates between 0.2 and 20 cm per year, with the global distribution of MOR opening rates^22^ (Fig. 4b). The resulting global model predicts an average crustal thickness of ∼7 km for full spreading rates higher than 5 cm per year and a total annual magma flux of 22.8 km^3^ per year (Fig. 4c), which matches observations^21^.

The increase in decompression melting in response to a falling sea level is more straightforward to calculate than for oceanic islands, as flexural effects do not need to be considered for the fully submerged global MOR system. We find that a 60 m sea level drop over 15 kyr transiently increases the average global magma production at MOR by 12% corresponding to 2.78 km^3^ per year (Fig. 4c).

### Impact on volcanic CO_2_ emissions and atmospheric imprint

To transfer the above estimates on extra decompression melting to increased volcanic CO_2_ emissions, assumptions regarding CO_2_ concentrations in the different mantle sources and possible time lags between enhanced melting and increased emissions have to be made. These assumptions are then used to evaluate the impact on atmospheric CO_2_, using a box model of the global carbon cycle.

**Figure 2:**
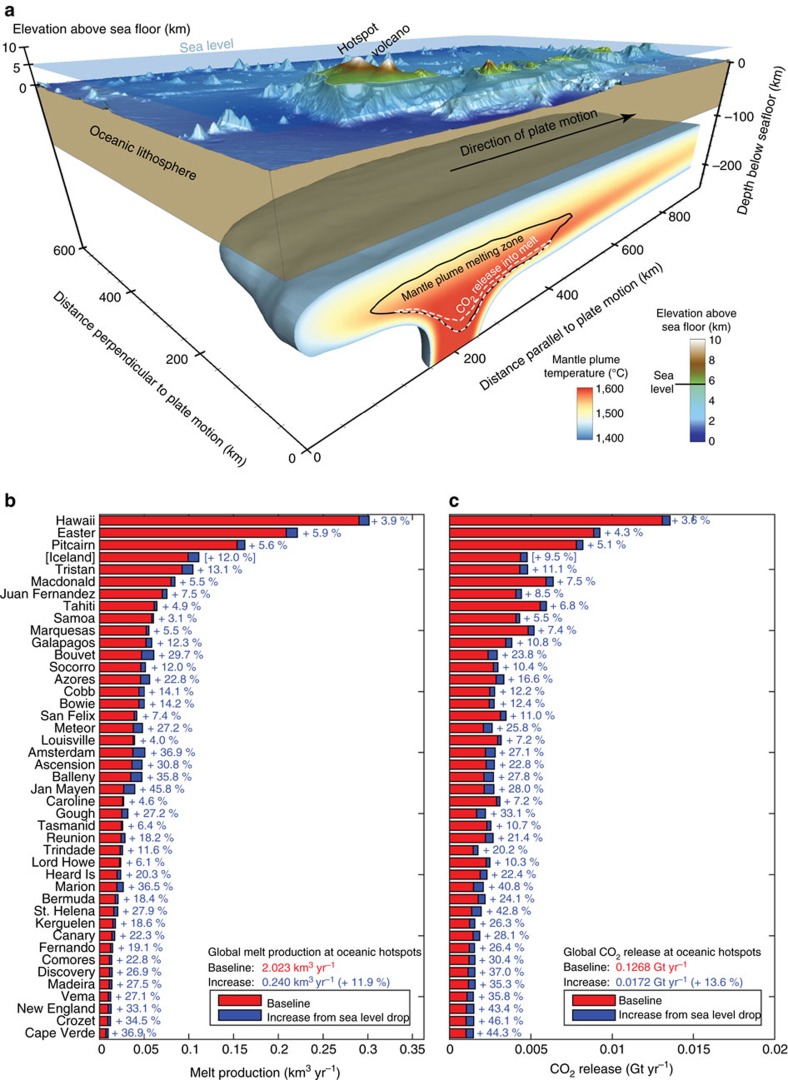
Effect sea level variations on hotspot melting and CO_2_ release. In (**a**) an example simulation for the Hawaiian plume (isosurface at T=1,420 °C) is shown. The magmatic system at depth extends beyond the plume stem and is susceptible to sea level changes. Size and shape of the melting region not only depend on the plume’s strength and composition but also on speed and age of the overlying lithosphere. The effect of a 60 m sea level drop over 15 kyr on magma flux (**b**) and CO_2_ release (**c**) is shown for the 43 oceanic hotspots investigated. Steady background values are shown in red, the increases during the MIS 5/4 sea level change in blue. Note that the melt and CO_2_ fluxes from the Iceland hotspot are only considered in the baseline but not in the calculation of the increase in response to a falling sea level. This has to been done to honour the observation that Iceland was glaciated during the last interglacial-glacial transition.

Carbon dioxide quantitatively degasses at MOR and hence its concentration in the mantle source is poorly known, with published values^23^ ranging from 50 to >2,000 p.p.m._w_. The average concentration is probably more on the lower side of this range^20^,^24^ and an average value of 140 p.p.m._w_ was estimated from ocean modelling of ^3^He and measured CO_2_/^3^He ratios^19^. We use this estimate and obtain a steady baseline CO_2_ flux of 0.096 Gt per year in our global MOR melting model (Fig. 4d), which, in spite of our more complex MOR melting model, is consistent with an earlier estimate^19^ and on the lower side of previously published estimates^25^ of 0.044–0.792 Gt CO_2_ per year. The average mantle CO_2_ concentration in the plume source is also poorly constrained. If CO_2_ is again assumed to behave similarly to He during partial melting, it can be inferred^20^ that the sources of ocean island basalts contain 120–1,830 p.p.m._w_ CO_2_. Here we use a value of 950 p.p.m._w_, which was determined from ^3^He as well as CO_2_/^3^He measurements and estimates of carbon-rich lower mantle contribution to plume melting^19^. Based on this we establish a baseline volcanic CO_2_ flux from oceanic hotpot volcanoes of 0.127 Gt CO_2_ per year (Fig. 2c). In correspondence, we predict that a 60 m sea level fall over 15 kyr results in an increase in the mobilization of CO_2_ into the melt phase of ∼14% (0.0172 Gt CO_2_ per year) beneath hotspot volcanoes and of ∼13% (0.0125 Gt CO_2_ per year) beneath the global MOR system. It should be noted that the uncertainty in these values is high—mainly because of the high uncertainty in the CO_2_ concentrations of the mantle source regions. As the scaling between the predicted increases in CO_2_ mobilization is linear with the CO_2_ concentration in the mantle source, the impact of different parameter choices can easily be assessed.

Further sensitivity tests assuming different magnitudes (60–100 m) and durations (5–15 kyr) of sea level fall show that the integrated amount of extra CO_2_ mobilization is on average 42 Gt CO_2_ per 10 m sea level drop at plume-related volcanoes and 31 Gt CO_2_ per 10 m sea level drop at MOR. This points to oceanic hotspots as the dominant geodynamic system with regard to sea level-linked variations in volcanic CO_2_ degassing. In total we predict ∼446 Gt of extra CO_2_ that was mobilized into the melt phase for the 60 m sea level drop throughout the MIS 5/4 boundary (Supplementary Table 3).

The climate relevance of these CO_2_ flux estimates will critically depend on how quickly CO_2_ is transported from the melting region to the atmosphere/ocean system and on how efficiently it is buffered on the release timescales by marine, terrestrial and atmospheric processes. There is now growing evidence that the magma flux at MORs can respond to sea level fall on suborbital timescales. Observational evidence for such a multi-millennial response time is the close relationship between sea level changes and hydrothermal activity that has been reported from both the South East Pacific Rise^6^ and the TAG hydrothermal on the Mid-Atlantic Ridge^7^. The reported time lags between change in sea level and a hydrothermal response, most likely driven by temporarily increased magmatic fluxes at these sites, are ∼5–15 kyr.

Relatively swift response timescales of such magmatic/hydrothermal systems to sea level changes within a few thousand years^7^ or less are in agreement with melt extraction timescales inferred from geochemical data. Analyses of uranium-series isotopic disequilibria point to minimum melt ascent rates of 1–20 m per year at MORs and oceanic hotspot volcanoes, and of ∼70 m per year at arc volcanoes, for which more stringent constraints are available^26^. Further support for rapid magma ascent comes from the large volumes of magma that were erupted within less than ∼2 kyr following the last deglaciation of Iceland, which implies melt ascent rates >50 m per year 27.

It thus seems that a magmatic response time to a sea level change in the order of a few kyr is fairly well supported by data. While it may seem intuitive that CO_2_ emission are likely to increase along with the magma flux, such a relation critically depends on details of the melt extraction process. CO_2_ is highly incompatible and partitions into the melt phase at the base of the melting column. Recent numerical modelling has shown that under steady-state conditions extra CO_2_ mobilized in response to a falling sea level may take several tens of kyr to reach the ridge axes^28^. This time lag is, however, likely to be smaller when transient effects and processes related to intermediate magma storage in, for example, the axial melt lens at MOR or magma chambers at hotspot volcanoes are considered. It should further be mentioned here that the timescales of melt extraction also affect the relative sensitivity of crustal production at MOR with differing spreading rates to different frequencies in the sea level forcing^2^. This relation introduces a ‘band-pass’ filter that controls how much of the sea level induced extra melt production reaches the ridge axis throughout a forcing period. This additional complexity is not considered here and we further take the simplifying view that an increase in magma flux is also related to an increase in the CO_2_ flux and will investigate the possible climate imprint of the predicted CO_2_ flux under the assumption that melt extraction occurs within a few thousand years.

Based on these assumptions we can evaluate the atmospheric impact of the additional volcanic CO_2_ release using the box model of the global carbon cycle BICYCLE^29^. We evaluate four scenarios (S1–S4) summarized in Supplementary Table 3 with different magnitudes (60–100 m) and durations (5–15 kyr) of the sea level drop with corresponding total additional volcanic CO_2_ releases of 400–697 Gt CO_2_. For simplicity, we assume in these carbon cycle simulations that all the CO_2_ is either injected into the atmosphere (subaerial) or into the deep ocean (submarine), corresponding to the release from hotspot volcanoes or MOR, respectively. For the subaerial carbon injection, the timing of the peak amplitude coincides with the end of the respective carbon injection in the different scenarios (Fig. 5a). A complete submarine source for the carbon release reduces the peak atmospheric CO_2_ value by 10–20% and the respective maximum is detected about 200 years later. After reaching the corresponding maximum values of 4–9 p.p.m._v_ atmospheric CO_2_ slowly decreases due to the carbonate compensation feedback of the sediments and the injected additional CO_2_ that stays in the atmosphere (the so-called airborne fraction) converges to about ∼6% on a 20 kyr timescale (Fig. 5b).

To set our results into context with other carbon cycle model sensitivities it is instructive to compare the airborne fraction in our model with those obtained by other studies, for example, by a model-intercomparison^30^ (MIP) in which a standardized pulse response scenario has been investigated. In this scenario 100 Gt C (=367 Gt CO_2_) are injected into the atmosphere within 1 year. The carbon cycle response to this injection is a function of background climate. The injection has been performed in the MIP^30^ under modern (not shown) and pre-industrial conditions (experiment pre-industrial times (PI) in Fig. 5c), and the evolution of airborne CO_2_ has been investigated for a period of 1 kyr or less. We here extrapolate these results towards 20 kyr by an impulse response function derived from the analysis of an Earth system model^31^. These extrapolated MIP results can be compared with the airborne fraction as simulated for PI and for 83 kyr ago (experiment 83ka) conditions (Fig. 5c) using BICYCLE. We find that the airborne fractions of our BICYCLE simulations are at the lower end of the 2*σ*-uncertainty spread of the MIP results (Fig. 5c). While a direct transfer of the airborne fraction spread to our results is not trivial, this model-intercomparison shows that our carbon cycle box model gives a relatively strong damping of volcanic CO_2_ release. Hence, our simulated impact on atmospheric CO_2_ is likely to represent a lower bound. Other models, for example, those used in the pulse response analysis^30^,^31^ would most likely find CO_2_ anomalies connected with this sea level induced volcanic outgassing that are up to a factor of 2.5 higher than our simulated peak responses (Fig. 5a).

## Discussion

Assuming a hypothetical baseline given by the simplifying assumption that orbital-scale CO_2_ concentration should have continued its close long-term correlation with temperature across the MIS 5/4 transition, the maximum CO_2_ offset in ice cores is as much as ∼18 p.p.m._v_ (Supplementary Fig 1a). Interestingly, our carbon cycle modelling results (Fig. 5a,b) suggest that enhanced volcanic degassing can explain the bulk of this disconnect or even the full offset if the uncertainties in airborne fractions are taken into account (Fig. 5c). The exact net effect, however, from the here presented negative feedback mechanism via hotspot and MOR volcanism will need to be further evaluated in the context of possibly counteracting processes such as carbonate formation during seafloor alteration reactions^32^,^33^ and decreased terrestrial volcanic emissions^1^,^34^.

Statistical analyses of the Quaternary eruption record do reveal a peak in activity at glaciated high-latitude volcanoes during the last deglacial, which is absent at volcanoes not affected by ice-sheet growth^1^,^35^. This suggests that growing ice-sheets during the MIS 5/4 transition may have resulted in reduced emissions at high-latitude volcanoes; especially since the regional loading effect can be much larger than the global decrease in sea level. Constraining the net effect and the respective roles that are played by arc, MOR and hotspot volcanoes in the Earth System is further complicated by the uncertainties in time lags between sea level as well as ice sheet forcing, and the different volcanic responses as discussed. These issues necessitate future research. This should include constraints refined by observations, improved multi-phase melt migration simulations, and coupled Earth system models that can account for volcanism-related solid Earth–climate interactions as possible causes for atmospheric CO_2_ changes.

The decoupling in the evolution of CO_2_ and temperature across the MIS 5/4 transition ended between ∼74 and 70 kyr ago when CO_2_ also started to drop (Fig. 1, Supplementary Fig. 1a). Available reconstructions indicate that sea level (Fig. 1f) is likely to have declined further beyond this point in time, which according to our suggested mechanism implies a sustained source of volcanic CO_2_ to the atmosphere. Hence, superposed changes in the atmosphere–ocean–biosphere system such as enhanced nutrient utilization in the Southern Ocean^29^,^36^ in response to increased dustiness^37^ leading to iron fertilization (Fig. 1e) and/or large scale changes in ocean circulation^14^,^38^,^39^ may have contributed to the overall CO_2_ decline and thus overprinted any further sea level/volcanism-related CO_2_ release after ∼74–70 kyr ago. The here proposed sea level-CO_2_ relation may have also, in addition or alternatively to previous explanations^40^, contributed to the persistence of stable high atmospheric CO_2_ concentrations at the end of the previous interglacial (MIS 5e) until ∼115 kyr ago, at a time when global long-term temperatures had already dropped for ∼10 kyr, approximately in tandem with falling sea level from high-stand conditions at ∼125 kyr ago (Fig. 1, Supplementary Fig 1b). Increasing uncertainty levels in the relative timing of atmospheric CO_2_, temperature and sea level changes provide a challenging framework to test the link between these changes and CO_2_ outgassing from volcanoes further back in time. Nevertheless, stacked records reveal that disconnects between CO_2_ and temperature during times of sea level fall are not unique to the MIS 5/4 transition (Supplementary Fig. 2). In contrast to the deglacial phases that show relatively synchronous changes in CO_2_ and temperature, most of the intervals of decreasing obliquity with falling sea level show a disconnect between CO_2_ and temperature for the last ∼800 kyr. Notable exceptions are phases of pronounced atmospheric dust variations, in line with our interpretation that superposed carbon cycle effects (via pronounced dust alterations or ocean circulation changes) can dominate over the impact of our presented sea level–degassing relation. This also applies to deglacial phases as suggested by the timing of pronounced atmospheric dust alterations (Supplementary Fig. 2). Furthermore the superposition of multiple carbon cycle effects within a relatively short deglacial period (∼10 kyr) also implies that this deglacial window might not be optimally suited to isolate geosphere–climate interactions such as the negative feedback mechanism described here. On longer time-scales (∼100 kyr), associated with the development of full interglacial-glacial changes, a sea-level drop of ∼120 m facilitates an atmospheric CO_2_ rise similar to the impact of a smaller glacial terrestrial carbon pool or a higher glacial ocean salinity^29^, which is expected to counteract the overall interglacial-glacial CO_2_ drawdown in the atmosphere via the negative feedback mechanism described here.

We conclude that throughout the last 800 kyr, the repeated occurrence of diverging trends in atmospheric CO_2_ levels and temperature during a synchronous sea level fall associated with ice sheet growth indicates that the proposed novel negative feedback mechanism (Supplementary Fig. 3) might be an important component of solid Earth–climate interactions and of the global carbon cycle on suborbital timescales.

## Methods

### Geodynamic modelling framework

All 2D and 3D geodynamic simulations have been conducted with the codes M2TRI and M3TET (refs 18, 41), respectively. These codes are written in MATLAB and solve for viscous incompressible flow, advection-diffusion of temperature, advection of composition and melting processes of mantle rocks that reach their pressure-, composition- and volatile-dependent solidus temperature.

### Governing equations and numerical methods

Mantle flow is described as viscous creep of an incompressible fluid (‘Stokes flow’) using the Boussinesq approximation, that is, density variations are only considered in the buoyancy term. We solve the equations for conservation of mass


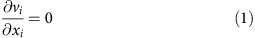


and momentum


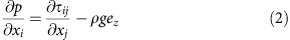


using the viscous stress–strain rate relationship


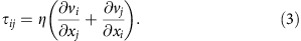


See Supplementary Table 4 for a complete list of variables and parameters used in the calculations. We assume a Newtonian rheology with the dynamic viscosity *η* depending on temperature *T* (in units of Kelvin), pressure *p* and weight fraction *χ*^H_2_O^ of water in the mantle rocks:


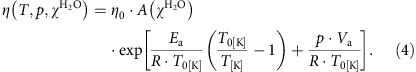


The pre-exponential factor *A* controls the viscosity increase during dehydration:


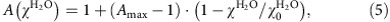


where *A*_max_ is the maximum increase in viscosity after complete dehydration. For numerical reasons we limit the viscosity variations to be between 3 × 10^18^ Pa s and 5 × 10^22^ Pa s. Density variations depend on temperature changes and the cumulative degree of melting *F*:


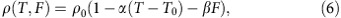


where *α* is the thermal expansion coefficient and *β* parameterizes the compositional buoyancy resulting from melting.

Energy conservation is formulated as


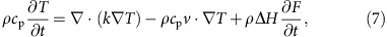


where Δ*H*=Δ*S*·*T* is the latent heat of fusion, and Δ*S* is the entropy of fusion.

We use the finite element method to solve for viscous flow and heat conduction. Advection of temperature and compositional fields are handled by the method of characteristics (also called semi-Lagrange method; for example, ref. 42). The matrix equation for the 2D Stokes flow problem is derived using the consistent penalty method combined with so-called Powell–Hestenes iterations (a formulation similar to that used in the MILAMIN code^43^). All matrix equations are solved using the Cholesky direct solver of the numerical library SuiteSparse^44^. In 3D, where the larger number of unknowns requires iterative solvers, we use a Schur-complement formulation^45^ to solve the velocity-pressure problem. This leads to an outer iteration to solve for pressure and an inner iteration updating the velocity field. For both iterations we use Conjugate Gradient (CG) solvers^46^, preconditioned by the pressure mass matrix (outer CG) and a single V-cycle of geometric multigrid (inner CG). M3TET is parallelized using MATLAB’s Parallel Computing Toolbox in combination with the MUTILS^47^ tools. For more details on the solution techniques see refs 18, 41.

The formulation to calculate melting rates in the mantle is based on ref. 48 and the reader is referred to this reference for the details of the melting model. We define solidus functions *T*^ *S*^ of the form


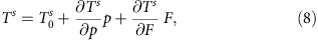


where 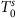 is the solidus temperature at surface pressure, 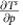 is the solidus dependence on pressure and 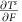 is the solidus dependence on depletion (Supplementary Table 4). We further consider wet melting and have implemented the parameterization described in ref. 49 into our framework by modifying the solidus-depletion gradient 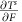 depending on the rock’s current water content. Water is treated like an incompatible chemical element that preferentially enters the melt phase. We use a partition coefficient *D*_H_2_O_=0.01 (ref. 49) and assume fractional melting, where the water content *X*^H_2_O^ of a mantle rock is calculated from its initial water content 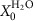 and cumulative degree of melting *F* (ref. 50)


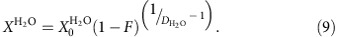


The solidus-depletion gradient is further modified to mimic the drop in melt productivity once clinopyroxene is exhausted from the residue (for example, ref. 51). For simplicity, we assume that this is the case for a degree of melting *F>*0.16 and increase 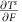 by a factor of 10 to reduce productivity (Supplementary Fig. 4).

### 2D mid-ocean ridge models

The numerical domain is 200 km deep and 400 km wide, and we assume symmetry along the ridge axis, that is, we model one half of the ridge. Temperature boundary conditions are 0 °C at the top and a potential mantle temperature *T*_M_*=*1,335 °C at the bottom inflow boundary. The symmetry axis (left side) and outflow boundary (right side) are thermally insulating. A constant half-spreading rate is prescribed along the top. Velocities along the inflow and outflow boundary are solved for in the first time step, with all buoyancy forces set to zero to avoid convective instabilities. From the second time step on velocities along the in- and outflow boundaries are fixed to these values and buoyancy forces are considered. We prefer this method for deriving the velocity boundary conditions over an analytical corner flow solution because the calculated in- and outflow velocities reflect the viscosity structure of the uppermost mantle. Calculations start with an initial temperature field calculated from the GDH1 plate model^52^ and we ran all calculations until a steady state in melt production was reached. Mantle composition and potential mantle temperature have been calibrated to produce a uniform crustal thickness of about 7 km at full spreading rates faster than 50 mm per year, in agreement with seismic observations^53^. We conducted a total of 360 2D numerical calculations in which we have varied (i) the half-spreading rate *v*_HS_ between 1 and 100 mm per year, (ii) the initial water content 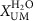 of the upper mantle (50 p.p.m._w_, 100 p.p.m._w_ and 200 p.p.m._w_; see ref. 54), and (iii) the maximum increase of the mantle rock viscosity during melting-related dehydration *A*_max_ (factor increase of 5, 10, 50 and 100). The value of the latter is somewhat uncertain so that we explored a broad range. The consequences of different parameter choices for the predicted global magma and CO_2_ mobilization rates are summarized in Supplementary Table 5. The global MOR model presented in the main text and in Fig. 4 is based on 2D simulations that assume 100 p.p.m._w_ water in the mantle source and a viscosity increase of factor 50 during melting-induced dehydration.

### 3D plume geodynamic models

Modelling the ascent and melting of a mantle plume beneath a moving lithospheric plate requires a 3D geodynamic model. We assume symmetry across a vertical plane parallel to the direction of plate motion and through the center of the plume, that is, we model one half of the plume. The numerical domain extends 400 km into the direction perpendicular to plate motion, 1,000 km into the direction of plate motion, and to 400 km depth. To have a precise control on the thickness of the overriding lithosphere we have excluded the rigid lithospheric plate from the model domain. Instead, the top of the domain corresponds to the base of the lithosphere and plume upwelling stops when this boundary is reached. Accordingly, we use a plate age-dependent heat flux boundary condition at the top to mimic the heat flow into the overlying lithosphere. A constant plate speed is prescribed along the top. A potential mantle temperature *T*_M_=1,335 °C is prescribed at the inflow boundary (left wall) upstream of the plume, all other side boundaries are insulating. The horizontal inflow velocities through the left wall are important because they create the ‘mantle wind’ through which the plume ascends. Here we initialize a viscosity-dependent vertical Couette flow profile with plate speed at the top and zero-velocity at 400 km depth. Free horizontal outflow is allowed through the right boundary downstream of the plume, while front and back boundaries are symmetric. Along the bottom we prescribe *T*_M_=1,335 °C except for a half-circular plume inflow region with 100 km radius, where we define a Gaussian-shaped temperature anomaly and a parabolic inflow profile. We control the plume strength by prescribing its excess temperature and buoyancy flux. Inflow velocities are then adjusted such that the desired buoyancy flux is exactly matched for the plume’s thermal structure. Depending on buoyancy and rheology the plume self-adjusts its radius well before reaching the melting region. All calculations ran until the plume head had passed the melting zone and a steady melt production established. We used the best-studied mantle plume/hotspot system *Hawaii* to calibrate the mantle composition for the mantle plume parameter study: For a plume buoyancy flux *Q*_B_=8,700 kg s^−1^ (ref. 55) and an excess temperature *T*_exc_=290 °C (ref. 56) we aimed to achieve an onset of melting at ∼170 km depth consistent with the seismically inferred melt-rich zone above this depth^57^. The total melt production below to the ∼91 Myr ago old lithosphere^58^ of ∼84 km thickness^52^ should be about 10 m^3^ s^–1^ (ref. 59). Meeting these constraints required a more fertile and wetter composition for mantle plume material 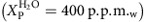 compared to the ‘upper mantle’ composition 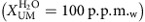. We conducted a 3D parameter study in which we systematically varied four key parameters related to the mantle plume and the plate tectonic setting of the associated hotspot: (i) plume buoyancy flux *Q*_B_ (500, 1,000, 4,000, 6,000 and 10,000 kg s^−1^), (ii) plume excess temperature *T*_exc_ (100, 200 and 300 °C), (iii) lithosphere thickness *h*_L_ at the hotspot location (50, 70 and 90 km), and (iv) plate speed *v*_L_ relative to the hotspot (10, 40 and 80 mm per year). The resulting 126 3D calculations span a 4D parameter space (Supplementary Fig. 5), within which the 43 oceanic hotspots (see Supplementary Table 2) have been linearly interpolated to derive their melt production and CO_2_ release rates. The advantage of the parameter-space approach is that it allows us to tests how critically the predicted global magma fluxes and CO_2_ mobilization rates depend on the four parameters that characterize each mantle plume/hotspot. The results of these sensitivity tests with the global plume model are summarized in Supplementary Table 6.

### CO_2_ mobilization and global mid-ocean ridge melting model

Assuming fractional melting^50^, the rate of CO_2_ mobilization into the melt phase in each model calculation was calculated using an initial concentration of 140 p.p.m._w_ CO_2_ (ref. 19) and a partition coefficient of *D*_CO_2__=0.01. Note the latter is a very moderate estimate since CO_2_ is likely to behave more incompatible than water, for which we use the same value. Each numerical calculation provides a rate of CO_2_ mobilization into the melt phase per metre ridge axis for a particular spreading rate. The estimate for the total CO_2_ mobilization along the global MOR system is derived using the global distribution of spreading rates^22^. Note that our global estimates (Fig. 4 and Supplementary Table 3) are in very good agreement with independent estimates for global magma production and CO_2_ release at MORs^19^. Melting beneath MOR results primarily from the decompression of mantle rocks. The enhanced CO_2_ mobilization into the melt phase at times of dropping sea level is calculated by superimposing the additional sea level-induced decompression onto the decompression of mantle rocks during upwelling. For a given amplitude Δ*h*_SL_ in sea level drop over a time span Δ*t*_SL_ and a sea water density *ρ*_w_=1,030 kg m^−3^ we calculate the rate of pressure change 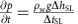 in the underlying mantle, which is then considered in the melting formulation.

### CO_2_ mobilization and global mantle plume melting model

For the global hotspot melting model we use a similar approach. The steady state solution of each 3D calculation is used to calculate the baseline melt production and CO_2_ mobilization into the melt phase, while the superimposed decompression during a prescribed sea level drop gives the enhanced values during ice sheet growth.

**Figure 3:**
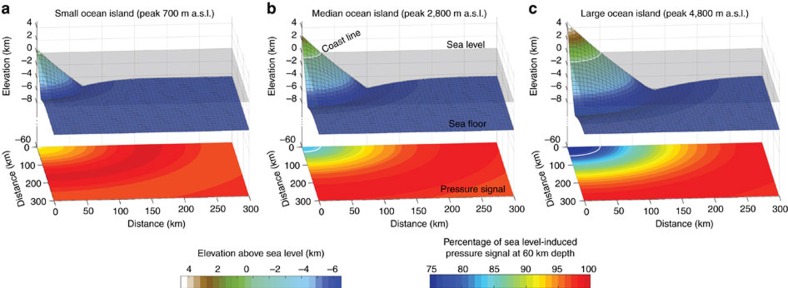
Results of 3D viscoelastic simulations that investigate the pressure signal beneath ocean islands of different size at a time of sea level fall. The upper panel show the different geometries of volcanic edifices supported by a viscoelastic lithosphere; the lower panel plots show the percentage of the pressure signal, induced by sea level change, that is present at 60 km depth in the asthenospheric mantle. A maximum damping of ∼25% is found for the largest island sizes. All considered hotspots were catagorized into small, medium and large island depending on their subaerial area (see Supplementary Table 2). (**a**) shows the results for a cone-shaped island with a maximum topography of 700 m, (**b**) for a medium island with 2,800 m topographic elevation, and (**c**) for a large island with 4,800 m elevation.

**Figure 4:**
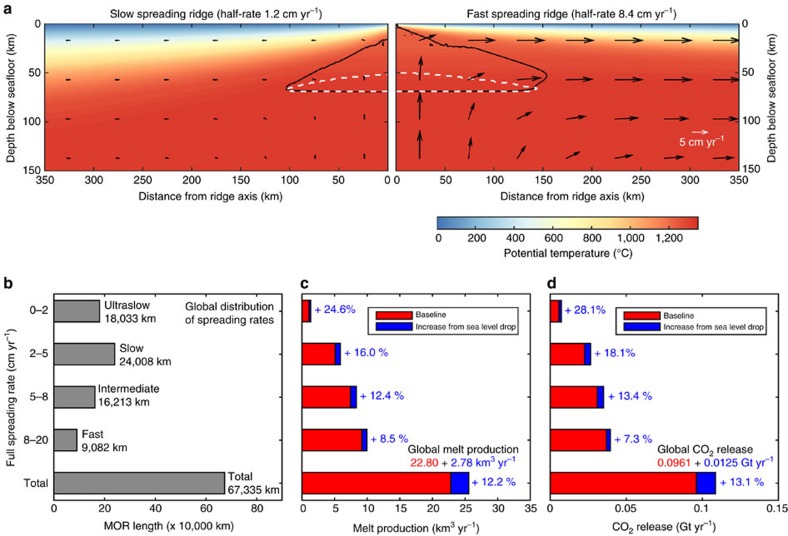
Effect of 60 m sea level drop over 15 kyr on MOR melting and CO_2_ release. (**a**) shows the temperature fields of a slow-and a fast-spreading ridge along with contours outlining the regions of pressure-release melting (black) and CO_2_ partitioning into the melt phase (white). (**b**) The global distribution of mid-ocean ridge opening rates^22^ is dominated by slow and ultraslow rates for which the relative importance of glacial sea level changes is highest. Computed increases in **c** magma and **d** CO_2_ fluxes during the MIS 5/4 sea level drop. Values are weighted with the global distribution of spreading rates shown in **b**. Red bars illustrate the steady background state and blue bars the increases associated with the 60 m sea level drop over 15 kyr.

**Figure 5:**
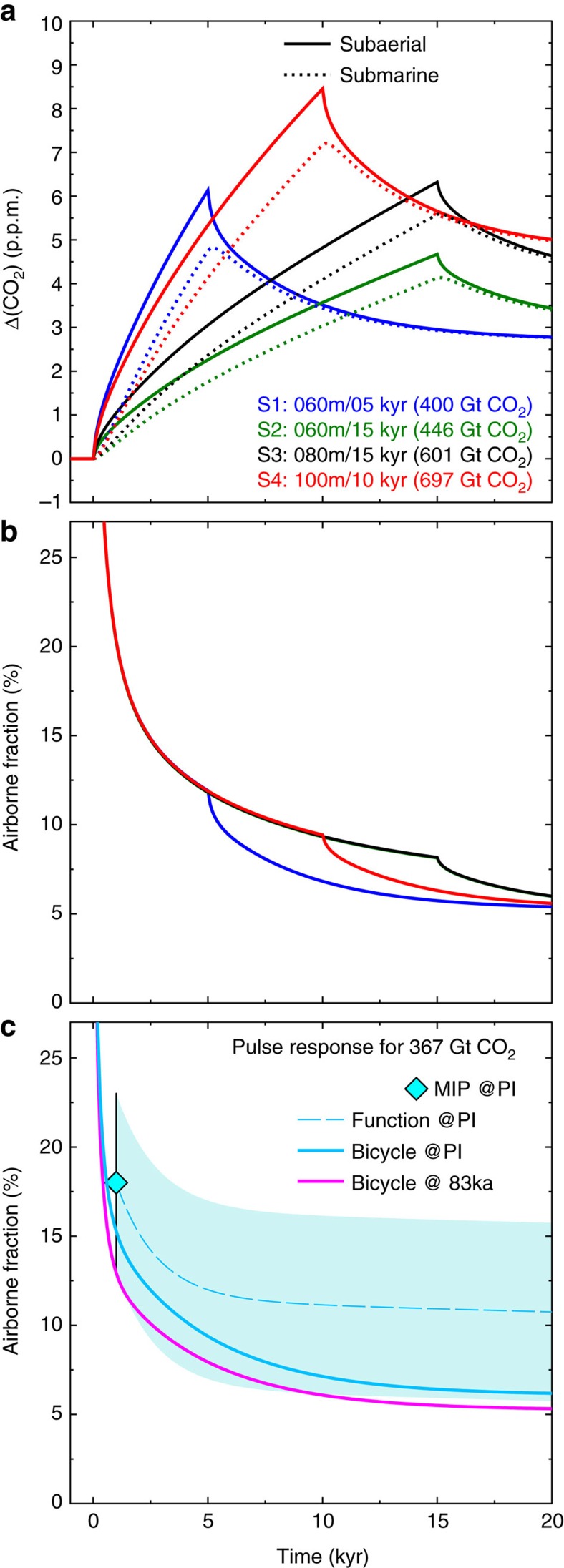
Impact of additional volcanic CO_2_ release in the atmosphere. Simulated changes in (**a**) atmospheric CO_2_ and (**b**) airborne fraction in response to the additional volcanic CO_2_ release related to sea level fall, using the global carbon cycle model BICYCLE^29^. Shown are the simulated anomalies for four different scenarios (S1-S4) in a setup in which the atmosphere-ocean sub-system of the carbon cycle is perturbed, including the sediment response (carbonate compensation). In all scenarios, the additional volcanic CO_2_ corresponds to the sum of MOR and hotspot melting related to sea level fall, released at constant annual rates. All emissions are either injected into the atmosphere (subaerial) or in the deep ocean (submarine). The simulations are initialized with boundary conditions representative of 83 kyr ago that are held constant throughout the experiments. In (**c**) the airborne fraction for a pulse response experiment of 367 Gt CO_2_ (=100 GtC) released in year 1 obtained with the BICYCLE model for background conditions of 83 kyr ago and of pre-industrial times (PI) are compared with PI results from a MIP^30^. MIP results are restricted to times up to 1 kyr, but are extrapolated (broken line) by results of a regression analysis (function) applied to the output of a Earth system model for a variety of emission scenarios^31^. The 2*σ*-uncertainty obtained from the MIP for 1 kyr is also used to show the likely uncertainty (model spread) for longer time periods (cyan-coloured area).

In contrast to the submarine MOR, oceanic hotspot melting zones are located below large volcanic edifices that are partially subaerial. A falling sea level, however, will only change the hydrostatic pressure on the seafloor. To quantify the amplitude of the sea level-induced pressure signal below the island we have calculated the viscoelastic response of an oceanic lithosphere carrying an island using the free surface and viscoelastic capabilities of M3TET. We initialized cone-shaped islands of different sizes, applied a water depth-dependent normal force boundary condition at the top and calculated the island’s subsidence until an isostatic equilibrium established (cf. Fig. 3). Then we simulated the falling sea level around the island by reducing the normal forces and monitored the pressure change in the viscous asthenospheric mantle. Elastic parameter choices are summarized in Supplementary Table 4. The pressure drop increases from a minimum value central below the island to the full sea level-induced signal at some distance to the island (Fig. 3). We have used these distance-to-island dependent pressure signals to calculate the enhanced melt production and CO_2_ mobilization rates in the plume melting region. For simplicity we have grouped all oceanic hotspots into four island-categories depending on their subaerial area (see Supplementary Table 2).

The derivation of global hotspot-related melt production and CO_2_ mobilization rates is more complicated than for MOR because more parameters control the melting of a particular hotspot. The first parameter is the plume buoyancy flux, where we use the values given in ref. 55 and in ref. 60. The second parameter is the plume excess temperature, which is based on ref. 56. Unfortunately, not every plume with estimated buoyancy flux also has an estimate for excess temperature and vice versa. To fill these data gaps we calculate the missing value using a linear interpolation between Hawaiian-like values (8,700 kg s^−1^ and 290 °C) representing the ‘strong plume’ case and values that are typical for a weak plume (500 kg s^−1^ and 150 °C). In case that both excess temperature and buoyancy flux are unconstrained we assume a weak plume with 500 kg s^−1^ and 130 °C. The 3rd and 4th parameters are speed and age of the lithosphere at the hotspot location, respectively. We use the hotspot locations given in ref. 60 and the NUVEL-1A model^61^,^62^ to first identify the lithospheric plate hosting the hotspot. The plate motion relative to the hotspot is then calculated using the global plate motion model described in ref. 63. Lithosphere thickness is calculated from the age of the lithosphere at the hotspot location (using the data set in ref. 58) and then converted to plate thickness using the GDH1 model^52^ for the thermal evolution of oceanic lithosphere. We define the lithosphere-asthenosphere boundary at the depth where 90% of the basal temperature in the GDH1 model is reached. Following the argumentation in ref. 64 we assume a compositional lithosphere of 50 km thickness that forms during melting and dehydration of mantle rocks at mid-ocean ridges. Hence, we assume a minimum plate thickness of 50 km even if the GDH1 model predicts a thinner thermal lithosphere. Using these four parameters, each oceanic hotspot/plume (Supplementary Table 2) is located within the 4D parameter space spanned by the 3D calculations (Supplementary Fig. 5), and melt production and CO_2_ mobilization are linearly interpolated. This approach had the advantage that we were able to test different estimates for plume buoyancy fluxes given, for example, in refs 55, 65 and to test how critically the predicted global fluxes depend on each of the four parameters. The results of these tests, summarized in Supplementary Table 6, show that the baseline fluxes are especially sensitive to variations in plume excess temperature and lithosphere thickness. The relative increase in global magma and CO_2_ flux during sea level drop are, nonetheless, very robust and do not significantly change.

### Carbon cycle simulations

The Box model of the Isotopic Carbon CYCLE–BICYCLE—is used to assess the impact of volcanic emissions on atmospheric CO_2_ concentrations. BICYCLE has been widely used for a broad range of Pleistocene paleoclimate applications^29^. BICYCLE is a model of the atmosphere–ocean–terrestrial biosphere-part of the global carbon cycle, in which the response of the deep ocean sediments (carbonate compensation feedback) to any change in the carbon cycle, including an external CO_2_ flux, is estimated with a pulse response function. It contains one homogeneous mixed atmosphere box, ten boxes in the ocean and seven boxes on land. The ocean is separated in surface, intermediate and deep waters in the Atlantic, Southern Ocean and Indo-Pacific. We used the BICYCLE model to assess the variations in atmospheric CO_2_ resulting from injecting volcanic CO_2_ into the model, either subaerial directly into the atmosphere or submarine into the water column. For these volcanic CO_2_ release experiments anomalies in atmospheric CO_2_ are calculated based on the model set-up as previously published^29^. Carbon cycle simulations are run for 7 kyr into quasi–equilibrium for climate conditions found 83 kyr ago leading to a simulated atmospheric CO_2_ concentration of 227 p.p.m._v_ for a set-up of atmosphere–ocean including carbonate compensation feedback. The volcanic CO_2_ release scenarios will be analysed as anomalies in this setup.

In detail, and based on the geodynamic modelling results, four carbon cycle scenarios have been simulated. In these simulations all boundary conditions are fixed at values for 83 kyr ago. In scenario S1 a sea level drop of 60 m over 5 kyr releases a total of 400 Gt CO_2_ (188 Gt CO_2_ from MOR+212 Gt CO_2_ from hotspots) corresponding to an annual flux of 80 Tg CO_2_. In S2 a sea level drop of 60 m over 15 kyr releases a total of 446 Gt CO_2_ (188 CO_2_ from MOR+258 Gt CO_2_ from hotspots) corresponding to an annual flux of 30 Tg CO_2_. In S3 a sea level drop of 80 m over 15 kyr releases a total of 601 Gt CO_2_ (252 CO_2_ from MOR+349 Gt CO_2_ from hotspots) corresponding to an annual flux of 40 Tg CO_2_. In scenario S4 a sea level drop of 100 m over 10 kyr releases a total of 697 Gt CO_2_ (314+383 Gt CO_2_ from hotspots) corresponding to an annual flux of 70 Tg CO_2_.

### Code availability

The M3TET and M2TRI codes are co-owned by Hamburg University, GEOMAR as well as Royal Holloway and the source code is not freely distributed. To help with all questions concerning reproducibility of the numerical results, further details on the used numerical implementations as well as data analysis procedures, additional data files and possibly precompiled sub-functions can be requested from the first author.

### Data availability

All relevant geodynamic modelling data are available from the first author. The carbon cycle modelling data are available at Pangaea with the doi: 10.1594/PANGAEA.874815.

## Additional information

**How to cite this article:** Hasenclever, J. *et al*. Sea level fall during glaciation stabilized atmospheric CO_2_ by enhanced volcanic degassing. *Nat. Commun.* **8**, 15867 doi: 10.1038/ncomms15867 (2017).

**Publisher’s note:** Springer Nature remains neutral with regard to jurisdictional claims in published maps and institutional affiliations.

## Supplementary Material

Supplementary Information

## References

[b1] Huybers P. & Langmuir C. Feedback between deglaciation, volcanism, and atmospheric CO_2_. Earth Planet. Sci. Lett. 286, 479–491 (2009).

[b2] Crowley J. W., Katz R. F., Huybers P., Langmuir C. H. & Park S.-H. Glacial cycles drive variations in the production of oceanic crust. Science 347, 1237–1240 (2015). 25766231 10.1126/science.1261508

[b3] Lund D. C. & Asimow P. D. Does sea level influence mid-ocean ridge magmatism on Milankovitch timescales? Geochem. Geophys. Geosyst. 12, Q12009 (2011).

[b4] Olive J.-A. . Sensitivity of seafloor bathymetry to climate-driven fluctuations in mid-ocean ridge magma supply. Science 350, 310–313 (2015). 26472905 10.1126/science.aad0715

[b5] Tolstoy M. Mid-ocean ridge eruptions as a climate valve. Geophys. Res. Lett 42, 1346–1351 (2015).

[b6] Lund D. . Enhanced East Pacific Rise hydrothermal activity during the last two glacial terminations. Science 351, 478–482 (2016). 26823422 10.1126/science.aad4296

[b7] Middleton J. L., Langmuir C. H., Mukhopadhyay S., McManus J. F. & Mitrovica J. X. Hydrothermal iron flux variability following rapid sea level changes. Geophys. Res. Lett. 43, 3848–3856 (2016).

[b8] Huybers P. & Langmuir C. H. Delayed CO_2_ emissions from mid-ocean ridge volcanism as a possible cause of late-Pleistocene glacial cycles. Earth Planet. Sci. Lett. 457, 238–249 (2017).

[b9] Barnola J. M., Raynaud D., Korotkevich Y. S. & Lorius C. Vostok ice core provides 160,000-year record of atmospheric CO_2_. Nature 329, 408–414 (1987).

[b10] Auffret G. A. . Record of hydrothermal activity in sediments from the Mid-Atlantic Ridge south of the Azores. Comptes Rendus De L Acad. Sci. Serie Ii Fascicule a-Sci. De La Terre Et Des Planet. 323, 583–590 (1996).

[b11] Frank M. . Beryllium 10, thorium 230, and protoactinium 231 in Galapagos microplate sediments—implications of hydrothermal activity and paleoproductivity changes during the last 100,000 years. Paleoceanography 9, 559–578 (1994).

[b12] Fischer H. . The role of Southern Ocean processes in orbital and millennial CO_2_ variations—a synthesis. Quarter. Sci. Rev. 29, 193–205 (2010).

[b13] Brovkin V., Ganopolski A., Archer D. & Munhoven G. Glacial CO_2_ cycle as a succession of key physical and biogeochemical processes. Clim. Past 8, 251–264 (2012).

[b14] Barker S. & Diz P. Timing of the descent into the last Ice Age determined by the bipolar seesaw. Paleoceanography 29, 489–507 (2014).

[b15] Grant K. M. . Rapid coupling between ice volume and polar temperature over the past 150,000 years. Nature 491, 744–747 (2012). 23151478 10.1038/nature11593

[b16] Bintanja R., van de Wal R. S. W. & Oerlemans J. Modelled atmospheric temperatures and global sea levels over the past million years. Nature 437, 125–128 (2005). 16136140 10.1038/nature03975

[b17] Medina-Elizalde M. A global compilation of coral sea-level benchmarks: implications and new challenges. Earth Planet. Sci. Lett. 362, 310–318 (2013).

[b18] Hasenclever J., Morgan J. P., Hort M. & Rüpke L. H. 2D and 3D numerical models on compositionally buoyant diapirs in the mantle wedge. Earth Planet. Sci. Lett. 311, 53–68 (2011).

[b19] Marty B. & Tolstikhin I. N. CO_2_ fluxes from mid-ocean ridges, arcs and plumes. Chem. Geol. 145, 233–248 (1998).

[b20] Dasgupta R. & Hirschmann M. M. The deep carbon cycle and melting in Earth's interior. Earth Planet. Sci. Lett. 298, 1–13 (2010).

[b21] Crisp J. A. Rates of magma emplacement and volcanic output. J. Volcanol. Geotherm. Res. 20, 177–211 (1984).

[b22] Bird P. An updated digital model of plate boundaries. Geochem. Geophys. Geosyst. 4, doi:; DOI: 10.1029/2001gc000252 (2003).

[b23] Saal A. E., Hauri E. H., Langmuir C. H. & Perfit M. R. Vapour undersaturation in primitive mid-ocean-ridge basalt and the volatile content of Earth's upper mantle. Nature 419, 451–455 (2002). 12368848 10.1038/nature01073

[b24] Hirschmann M. M. & Dasgupta R. The H/C ratios of Earth's near-surface and deep reservoirs, and consequences for deep Earth volatile cycles. Chem. Geol. 262, 4–16 (2009).

[b25] Burton M. R., Sawyer G. M. & Granieri D. Deep carbon emissions from volcanoes. Rev. Mineral. Geochem. 75, 323–354 (2013).

[b26] Turner S. P. & Bourdon B. in Timescales of Magmatic Processes 102–115John Wiley & Sons, Ltd (2010).

[b27] MacLennan J., Jull M., McKenzie D., Slater L. & Gronvold K. The link between volcanism and deglaciation in Iceland. Geochem. Geophys. Geosyst. 3, doi:; DOI: 10.1029/2001gc000282 (2002).

[b28] Burley J. M. A. & Katz R. F. Variations in mid-ocean ridge CO_2_ emissions driven by glacial cycles. Earth Planet. Sci. Lett. 426, 246–258 (2015).

[b29] Köhler P., Fischer H. & Schmitt J. Atmospheric δ^13^CO_2_ and its relation to pCO_2_ and deep ocean δ^13^C during the late Pleistocene. Paleoceanography 25,, PA1213 (2010).

[b30] Joos F. . Carbon dioxide and climate impulse response functions for the computation of greenhouse gas metrics: a multi-model analysis. Atmos. Chem. Phys. 13, 2793–2825 (2013).

[b31] Lord N. S., Ridgwell A., Thorne M. C. & Lunt D. J. An impulse response function for the ‘long tail’ of excess atmospheric CO_2_ in an Earth system model. Global. Biogeochem. Cycles 30, 2–17 (2016).

[b32] Staudigel H., Hart S. R., Schmincke H. U. & Smith B. M. Cretaceous ocean crust at DSDP site-417 and site-418—Carbon uptake from weathering versus loss by magmatic outgassing. Geochim. Cosmochim. Acta 53, 3091–3094 (1989).

[b33] Alt J. C. & Teagle D. A. H. The uptake of carbon during alteration of ocean crust. Geochim. Cosmochim. Acta 63, 1527–1535 (1999).

[b34] Roth R. & Joos F. Model limits on the role of volcanic carbon emissions in regulating glacial-interglacial CO_2_ variations. Earth Planet. Sci. Lett. 329, 141–149 (2012).

[b35] Brown S. . Characterisation of the Quaternary eruption record: analysis of the Large Magnitude Explosive Volcanic Eruptions (LaMEVE) database. J. Appl. Volcanol. 3, 5 (2014).

[b36] Martinez-Garcia A. . Iron fertilization of the subantarctic ocean during the last Ice Age. Science 343, 1347–1350 (2014). 24653031 10.1126/science.1246848

[b37] Lambert F. . Dust-climate couplings over the past 800,000 years from the EPICA Dome C ice core. Nature 452, 616–619 (2008). 18385736 10.1038/nature06763

[b38] Thornalley D. J. R., Barker S., Becker J., Hall I. R. & Knorr G. Abrupt changes in deep Atlantic circulation during the transition to full glacial conditions. Paleoceanography 28, 253–262 (2013).

[b39] Bereiter B. . Mode change of millennial CO_2_ variability during the last glacial cycle associated with a bipolar marine carbon seesaw. Proc. Natl Acad. Sci. USA 109, 9755–9760 (2012). 22675123 10.1073/pnas.1204069109 PMC3382554

[b40] Brovkin V. . Comparative carbon cycle dynamics of the present and last interglacial. Quarter. Sci. Rev. 137, 15–32 (2016).

[b41] Hasenclever J. Modeling Mantle Flow and Melting Processes at Mid-Ocean Ridges and Subduction Zones — Development and Application of Numerical Models (PhD thesis, Hamburg University (2010).

[b42] Staniforth A. & Côté J. Semi-Lagrangian integration schemes for atmospheric models—a review. Mon. Weather Rev. 119, 2206–2223 (1991).

[b43] Dabrowski M., Krotkiewski M. & Schmid D. W. MILAMIN: MATLAB-based finite element method solver for large problems. Geochem. Geophys. Geosyst. 9, doi:; DOI: 10.1029/2007gc001719 (2008).

[b44] Davis T. A. & Hager W. W. Dynamic supernodes in sparse Cholesky update/downdate and triangular solves. Acm Transactions on Mathematical Software 35, doi:; DOI: 10.1145/1462173.1462176 (2009).

[b45] Mayday Y. & Patera A. T. in *State of the Art Surveys on Computational Mechanics* (eds Noor, A. K. and Oden, T. J.) 71–143American Society of Mechanical Engineers, New York (1989).

[b46] Saad Y. Iterative Methods for Sparse Linear Systems Society of Industrial and Applied Mathematics (2003).

[b47] Krotkiewski M. & Dabrowski M. Parallel symmetric sparse matrix-vector product on scalar multi-core CPUs. Parallel Comput. 36, 181–198 (2010).

[b48] Morgan J. P. Thermodynamics of pressure release melting of a veined plum pudding mantle. Geochem. Geophys. Geosyst. 2, (2001).

[b49] Katz R., Spiegelman M. & Langmuir C. H. A new parameterization of hydrous mantle melting. Geochem. Geophys. Geosyst. 4, 1073 (2003).

[b50] Langmuir C., Klein E. M. & Plank T. in Mantle Flow and Melt Generation at Mid-Ocean Ridges Geophysical Monograph eds Morgan J. P., Blackman D., Sinton J. American Geophysical Union (1992).

[b51] Hirschmann M. M., Ghiorso M. S., Wasylenki L. E., Asimow P. D. & Stolper E. M. Calculation of peridotite partial melting from thermodynamic models of minerals and melts. I. Review of methods and comparison with experiments. J. Petrol. 39, 1091–1115 (1998).

[b52] Stein C. A. & Stein S. A model for the global variation in oceanic depth and heat flow with lithospheric age. Nature 359, 123–129 (1992).

[b53] Bown J. W. & White R. S. Variation with spreading rate of oceanic crustal thickness and geochemistry. Earth Planet. Sci. Lett. 121, 435–449 (1994).

[b54] Hirth G. & Kohlstedt D. in Inside the Subduction Factory ed. Eiler John 83–105American Geophysical Union (2013).

[b55] Sleep N. H. Hotspots and mantle plumes—some phenomenology. J. Geophys. Res. 95, 6715–6736 (1990).

[b56] Putirka K. Excess temperatures at ocean islands: implications for mantle layering and convection. Geology 36, 283–286 (2008).

[b57] Rychert C. A., Laske G., Harmon N. & Shearer P. M. Seismic imaging of melt in a displaced Hawaiian plume. Nat. Geosci. 6, 657–660 (2013).

[b58] Muller R. D., Sdrolias M., Gaina C. & Roest W. R. Age, spreading rates, and spreading asymmetry of the world's ocean crust. Geochem. Geophys. Geosyst. 9, 19 (2008).

[b59] Van Ark E. & Lin J. Time variation in igneous volume flux of the Hawaii-Emperor hot spot seamount chain. J. Geophys. Res. Solid Earth 109, doi:; DOI: 10.1029/2003jb002949 (2004).

[b60] Steinberger B. Plumes in a convecting mantle: models and observations for individual hotspots. J. Geophys. Res. Solid Earth 105, 11127–11152 (2000).

[b61] Demets C., Gordon R. G., Argus D. F. & Stein S. Current plate motions. Geophys. J. Int. 101, 425–478 (1990).

[b62] Demets C., Gordon R. G., Argus D. F. & Stein S. Effect of recent revisions to the geomagnetic reversal timescale on estimates of current plate motions. Geophys. Res. Lett. 21, 2191–2194 (1994).

[b63] Morgan W. J. & Morgan J. P. Plate velocities in the hotspot reference frame. Geological Soc. Am. Special Papers 430, 65–78 (2007).

[b64] Phipps Morgan J. The generation of a compositional lithosphere by mid-ocean ridge melting and its effects on subsequent off-axis hotspot upwelling and melting. Earth Planet. Sci. Lett. 146, 213–232 (1997).

[b65] Davies G. F. Ocean bathymetry and mantle convection 1. Large-scale flow and hotspots. J. Geophys. Res. 93, 10467–10480 (1988).

[b66] Laskar J. . A long-term numerical solution for the insolation quantities of the Earth. Astron. Astrophys. 428, 261–285 (2004).

[b67] Kindler P. . Temperature reconstruction from 10 to 120 kyr b2k from the NGRIP ice core. Clim. Past 10, 887–902 (2014).

[b68] Jouzel J. . Orbital and millennial Antarctic climate variability over the past 800,000 years. Science 317, 793–796 (2007). 17615306 10.1126/science.1141038

[b69] Veres D. . The Antarctic ice core chronology (AICC2012): an optimized multi-parameter and multi-site dating approach for the last 120 thousand years. Clim. Past 9, 1733–1748 (2013).

[b70] Shakun J. D., Lea D. W., Lisiecki L. E. & Raymo M. E. An 800-kyr record of global surface ocean and implications for ice volume-temperature coupling. Earth Planet. Sci. Lett. 426, 58–68 (2015).

