# Supplementary material for: Sea level fall during glaciation stabilized atmospheric CO2 by enhanced volcanic degassing
Source: Nat Commun. 2017 Jul 6;8:15867. doi: 10.1038/ncomms15867 (PMC5504290; doi:10.1038/ncomms15867)
Supplement: Supplementary Information [file ncomms15867-s1.pdf]

Type of file: PDF
Size of file: 0 KB
Title of file for HTML: Supplementary Information
Description: Supplementary Figures, Supplementary Tables and Supplementary References

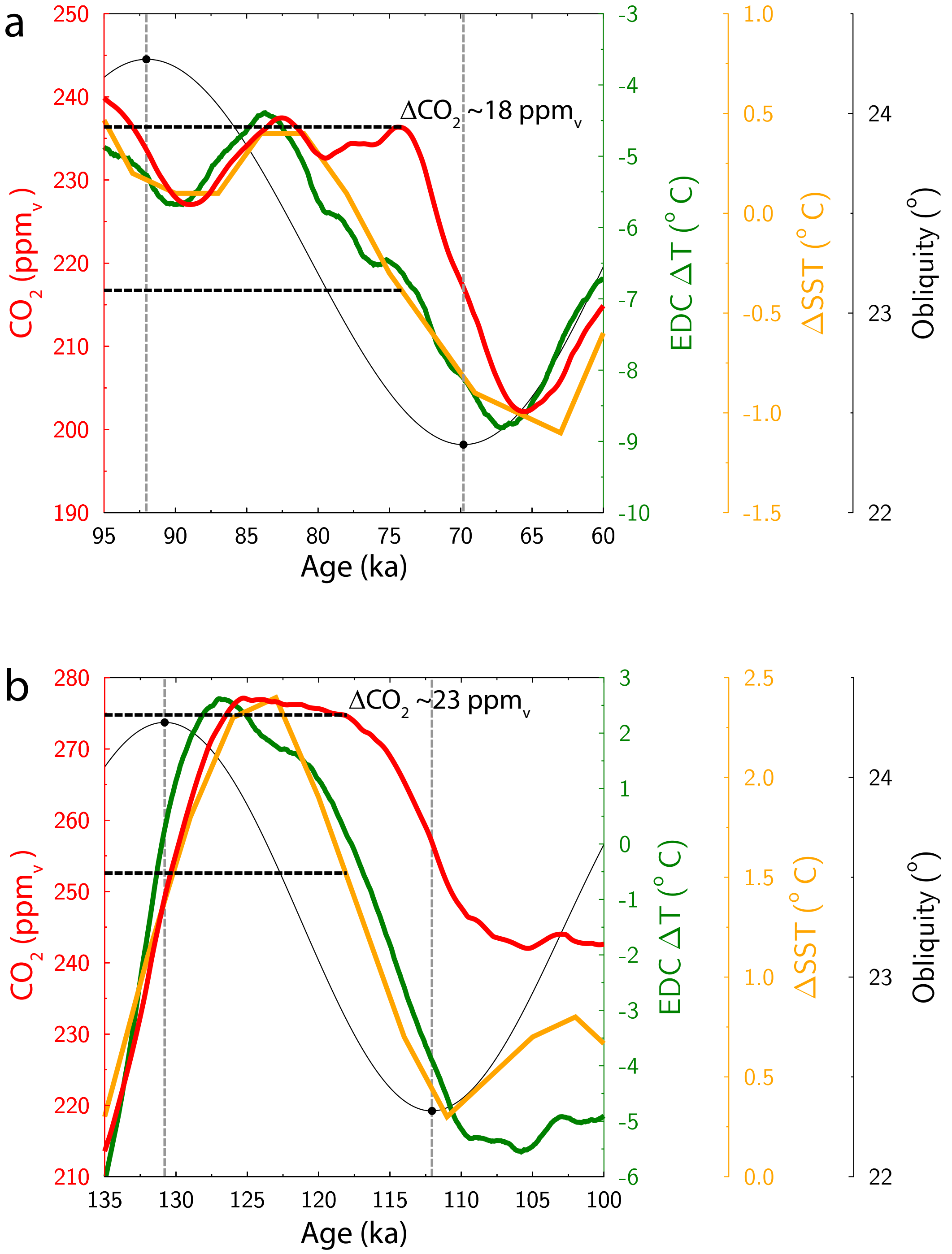

Supplementary Figure 1*: Data-based estimate of the amplitude of the disconnect between atmospheric CO_2 and temperature. Shown are changes in atmospheric CO_2, EDC temperature change, global SST anomaly, and obliquity during* ***(a)*** *the MIS 5/4 transition and* ***(b)*** *at the end of MIS 5e. Data are the same as in Figure 1 (ΔSST, obliquity and the 7-kyr-running mean in all other records). Vertical dashed lines denote the interval of decreasing obliquity. Horizontal dashed lines mark an upper limit of the CO_2-offset (ΔCO_2) associated with the decoupling of temperature and CO_2.*

Supplementary Figure 2

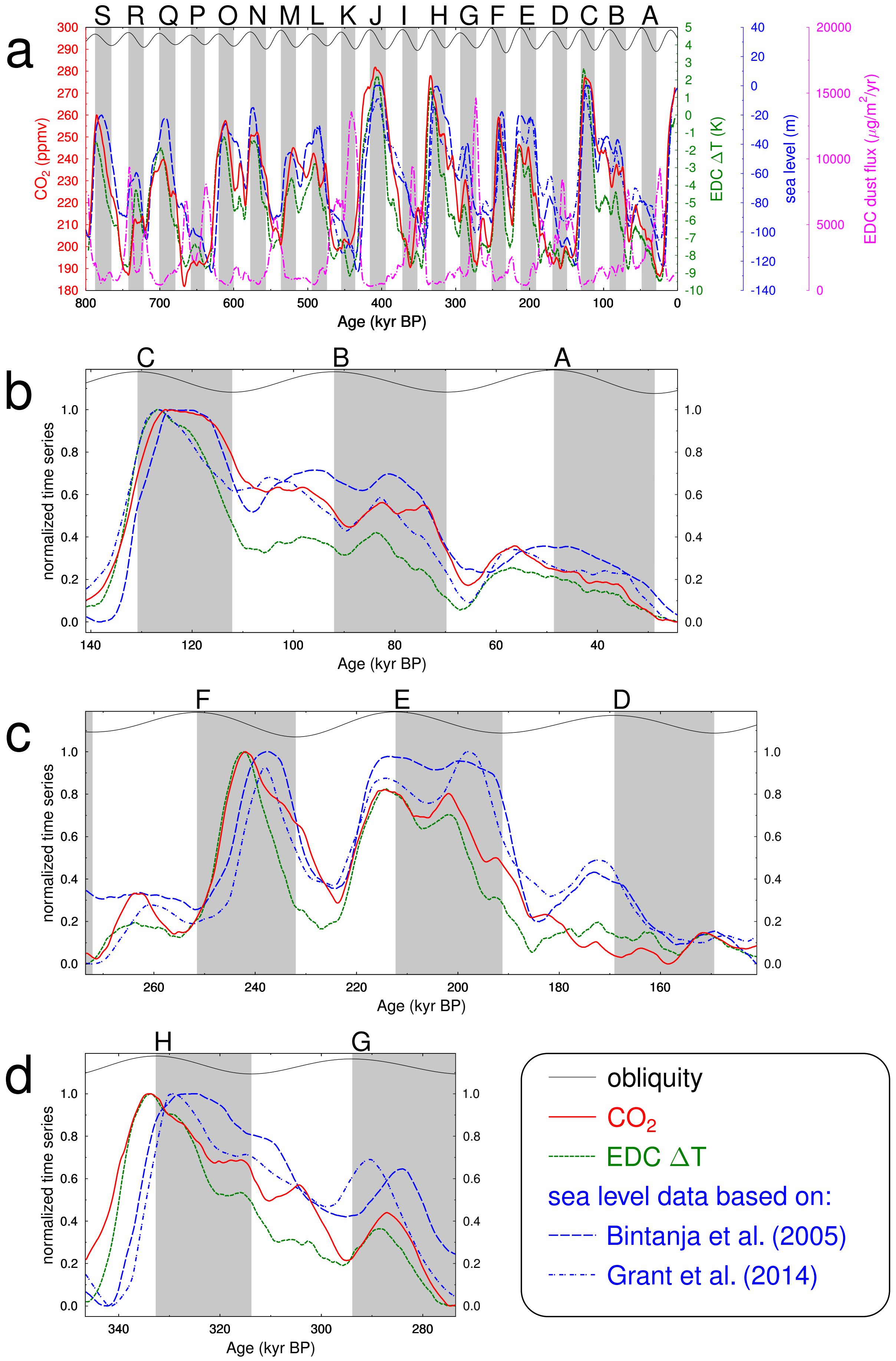

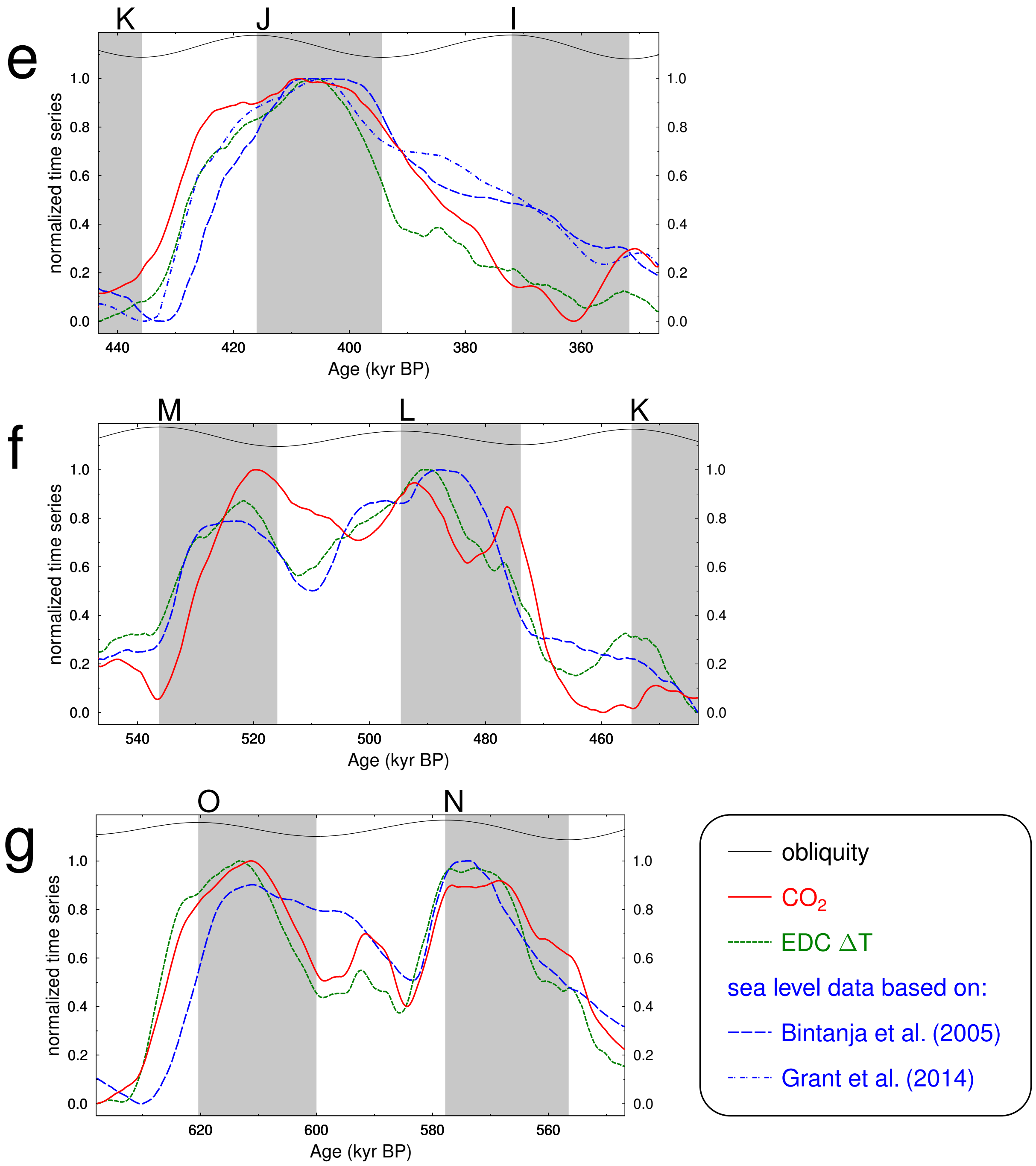

e
K
J
I
normalized time series
0.0
0.2
0.4
0.6
0.8
1.0
440
420
400
380
360
Age (kyr BP)
f
M
L
K
540
520
500
480
460
Age (kyr BP)
g
O
N
620
600
580
560
Age (kyr BP)
obliquity
CO_2
EDC ΔT
sea level data based on:
Bintanja et al. (2005)
Grant et al. (2014)

Supplementary Figure 2 (continued)

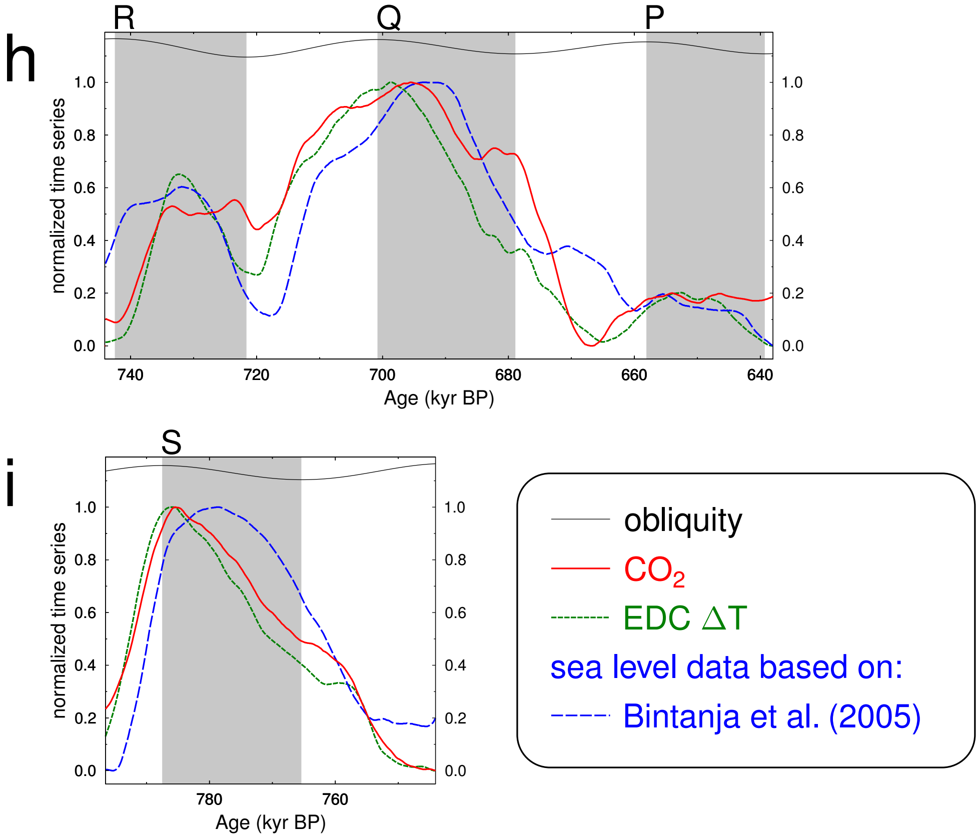

Supplementary Figure 2: *Changes in obliquity and different proxy-data records during the last 800 kyr.* ***(a)*** *Records for Antarctic (EDC) temperature*[1]*, atmospheric* CO_2[2]*, EDC dust flux*[3] *and two different estimates of sea level change (the 500 kyr-long Red Sea record of ref.*[4] *and model-based inversion of the LR04 benthic* $\delta^{18}O$ *record of ref.*[5] *during the last 800 kyr. The sea level reconstruction of ref.*[5] *representing the model-based inversion of the LR04 benthic* $\delta^{18}O$*, is shown as published, all others as 7 kyr running mean to reduce millennial-scale variability. All EDC records are shown on the most recent age model AICC2012*[6,7]*. The grey bars (labelled A-S) indicate periods with decreasing obliquity*[8]*, which is shown on top of each panel without y-axis. Normalized versions of EDC temperature,* CO_2 *and sea level are shown for the last eight glacial cycles in panels* ***(b-i)****. Each panel starts with a glacial maximum (minimum in EDC temperature) prior to a deglaciation and covers one glacial cycle until the next glacial maximum prior to the following deglaciation containing Termination II* ***(b)*** *up to Termination IX* ***(i).*** *In these panels the data for EDC temperature,* CO_2 *and sea level have been normalized by their total ranges contained in the respective glacial cycle. The normalized records show that the characteristics at the MIS 5/4 transition (cf. Fig. 1) are not unique. In contrast to the deglacial phases that show relatively synchronous changes in* CO_2 *and EDC temperature, most of the intervals of decreasing obliquity (A-S) with falling sea level show a disconnection between* CO_2 *and EDC temperature during the last eight glacial cycles* ***(b-i)****. Notable exceptions are phases of pronounced variations in EDC dust flux, e.g. at the end of interval B or interval D in panel* ***(a)****, which point to the importance of superposed dust alterations that can impact on atmospheric* CO_2 *via iron fertilization in the Southern Ocean*[9,10]*.*

Supplementary Figure 3

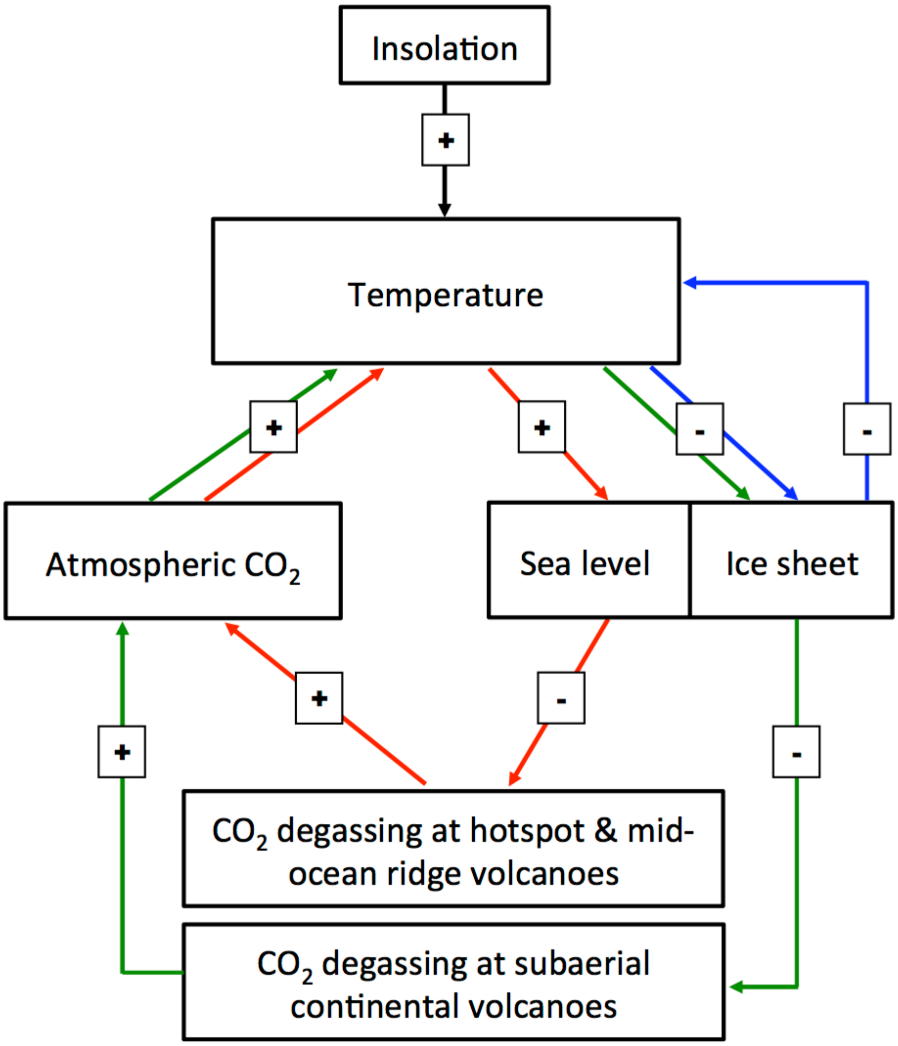

Supplementary Figure 3*: Idealized representation of the causal structure describing the influence of glaciation on atmospheric CO_2 and temperature in the context of solid earth-climate interactions. An encircled plus denotes a positive effect of one quantity onto another, a minus a negative one. In the negative feedback loop described in our study (red arrows), a sea level decline during ice sheet growth causes enhanced volcanic degassing of CO_2. Additionally two positive feedback loops associated with the blue and green arrows illustrate links that can be summarised as classical ice albedo feedbacks due to temperature and ice sheet changes (blue arrows), as well as the impact of volcanic degassing via subaerial continental volcanoes (green arrows) according to ref.[11]. Our results imply that during intervals of pronounced ice growth and sea level drop, the temporal evolution of temperature is dominated by e.g. ice albedo feedbacks (blue arrows), while the negative feedback loop described in our study (red arrows) counteracts the positive feedback loop (green arrows) and favours a stabilisation of atmospheric CO_2 levels.*

Supplementary Figure 4

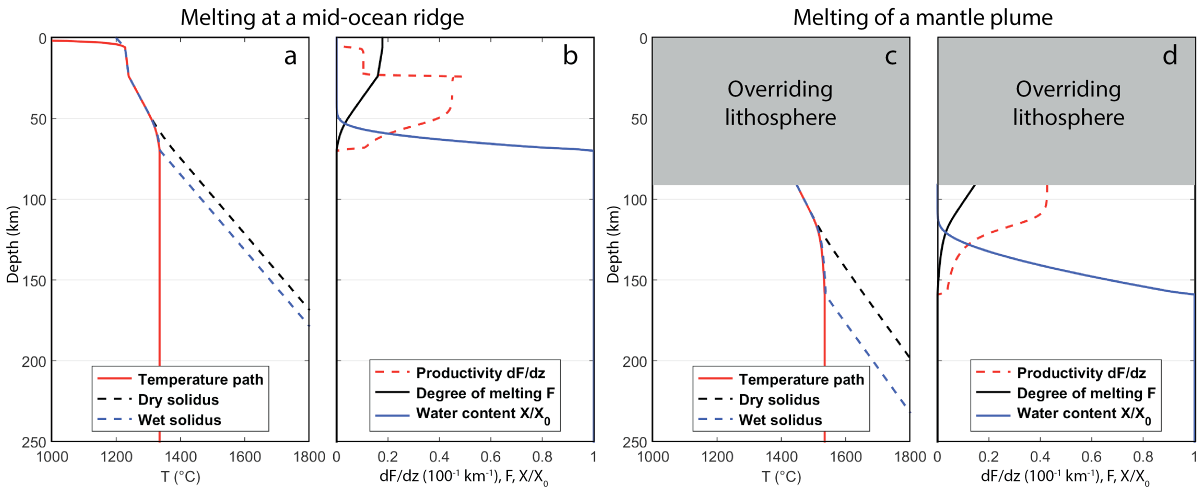

***Supplementary Figure 4**: Temperature paths (solid red) of the upper mantle rising adiabatically below a mid-ocean ridge (**a**, potential temperature of 1335 °C) and of a mantle plume rising below a 90 km thick lithospheric plate (**c**, potential temperature is 1535 °C). Wet and dry solidus functions are shown in dashed blue and dashed black, respectively, for both scenarios. See Methods and Supplementary Table 4 for the definition of the solidus functions. Panels **(b)** and **(d)** show melt production during upwelling (dF/dz, dashed red), the cumulative degree of melting F (solid black) and the water content X in the residue relative to the initial content X_0 (solid blue). We assume X_0=100 ppm$_w$ for the mid-ocean ridge model and X_0=400 ppm$_w$ for the mantle plume model.*

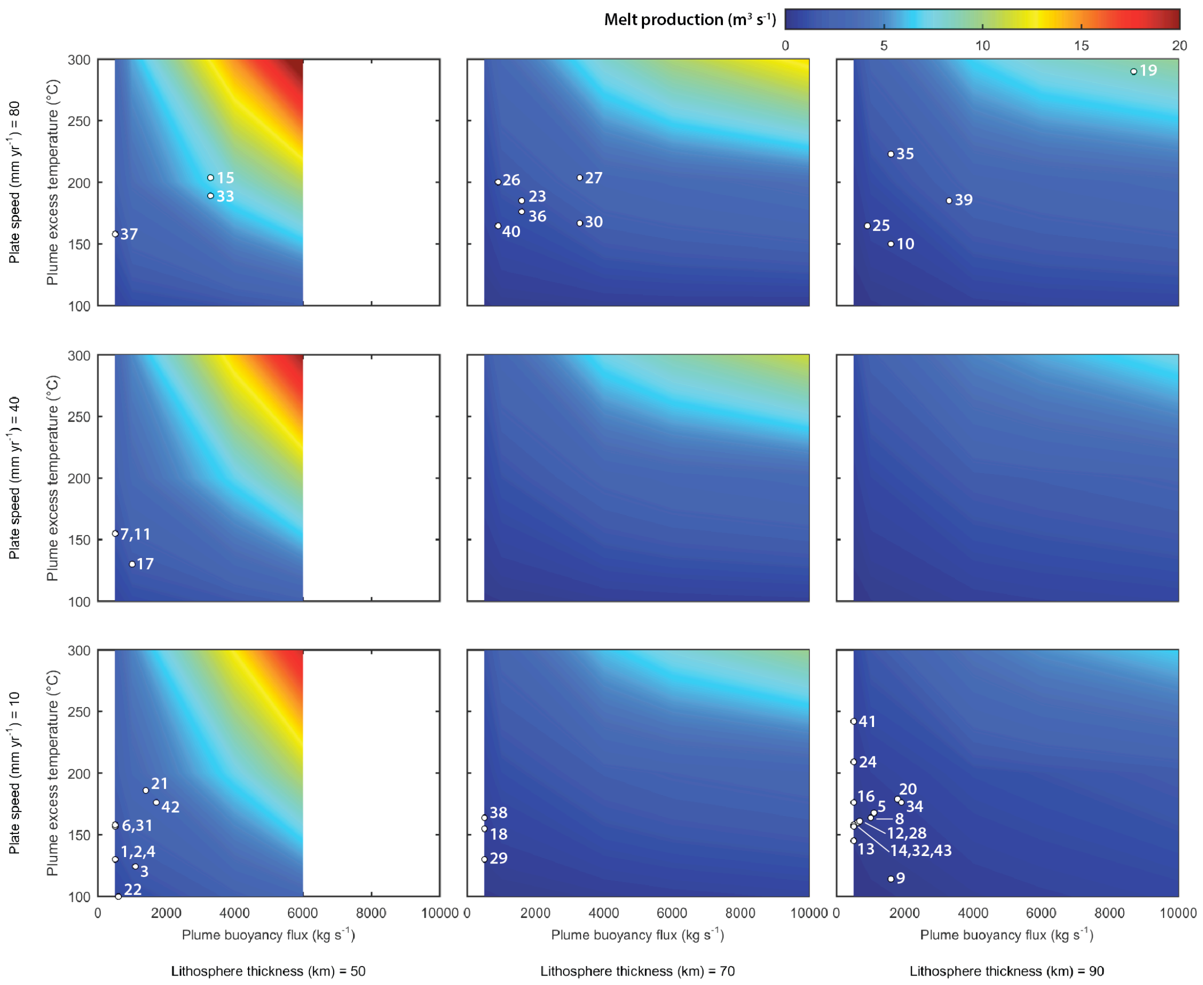

***Supplementary Figure 5**: Melt production in the four-dimensional parameter space that was constructed using 126 3-D model calculations. Melt production is shown as a function of lithosphere thickness (columns), plate speed relative to the hotspot (rows), plume buoyancy flux (x-axis in each panel), and plume excess temperature (y-axis in each panel). The 43 global hotspots are plotted as white dots within this parameter space and the attached numbers refer to each hotspot in Supplementary Table 2. For this illustration, each hotspot is plotted in the nearest row (plate speed) and column (lithosphere thickness) subplot. Note, however, that the values for magma and CO_2 fluxes have been calculated for each hotspot by a full interpolation in the four-dimensional parameter space. The best-studied and strongest global hotspot Hawaii (number 19) has been used to calibrate the mantle composition for all plumes (see Methods).*

		Sea level below present (m)					Range of sea level change (m)		
Reference		85 ka	80 ka	75 ka	70 ka		85 to 70 ka	80 to 70 ka	75 to 70 ka
Grant et al., 2012[13] 95 %	Max.<br>Avg.<br>Min.	67<br>50<br>31	67<br>52<br>32	79<br>63<br>42	94<br>81<br>64		-63<br>-31<br>+3	-62<br>-29<br>+3	-52<br>-18<br>+15
Bintanja et al., 2005[5] 2 sigma	Max.<br>Avg.<br>Min.	62<br>45<br>28	54<br>38<br>21	66<br>50<br>33	98<br>79<br>59		-70<br>-34<br>+3	-77<br>-41<br>-5	-65<br>-29<br>+7
Medina-Elizalde, 2013[12] 95 %	Max.<br>Avg.<br>Min.	*21*<br>16<br>**10**	14<br>9<br>4	48<br>34<br>20	**101**<br>74<br>*47*		**-91**<br>-58<br>*-26*	-97<br>-65<br>-33	-81<br>-40<br>+1

Supplementary Table 1*: Analysis of the different published sea level curves as shown in Fig. 1f of the main manuscript. This table lists the sea level values at 85, 80, 75 and 70 ka BP (the white columns in S1 contain sea level in meters below present with positive values representing a lower sea level; grey columns show the change in sea level for the corresponding time intervals with negative implying a sea level drop). The respective min. and max. values around the average values refer to the 95% probability envelopes. The corresponding sea level changes are calculated according to the following example: In the interval between 85-70 ka BP the average sea level change (-58 m) for Medina-Elizalde [12] is derived from the difference between the avg. sea level (74 m) at 70 ka and the avg. sea level (16 m) at 85 ka BP. The maximum sea level change (-91 m) is derived from the difference between the max. sea level (101 m) at 70 ka and the min. sea level (10 m) at 85 ka BP. Vice versa the min. sea level change (-26 m) is derived from the difference between the min. sea level (47 m) at 70 ka and the max. sea level (21 m) at 85 ka BP. Since this calculation combines minimum and maximum values of different rows in the white columns, the two examples have been marked by bold (maximum) and italics (minimum). For our baseline scenario S2 (60 m sea level decline in 15 kyrs, with 4.0 m kyr^{-1}), 9 of the 27 realisations shown in the table have a larger magnitude of sea level change (>60 m) and 13 realisations have a larger associated rate of sea level change (>4 m kyr^{-1}). The other sensitivity runs in our study (S1, S3, S4, shown in Supplementary Table 3) represent more pronounced scenarios with the largest sea level change in S4 (100 m sea level decline in 10 kyr, with 10 m kyr^{-1}) and the strongest rate of sea level change in S1 (60 m sea level decline in 5 kyr, with 12 m kyr^{-1}), which exploit the potential towards extremer changes shown in this table (e.g. 97 m sea level decline in 10 kyr, with 9.7 m kyr^{-1}; 81 m in 5 kyr sea level decline, with 16.2 m kyr^{-1}). It should be noted that plausible realisations in the table also include smaller and slower sea level drops than in our baseline scenario S2.*

Hotspot	ID	Lat	Long	Buoyancy flux (kg/s)	Excess temperature (K)	Plate speed (mm/yr)	Lithosphere age (Myr)	Lithosphere thickness (km)	Island category
Amsterdam	1	-38.0	77.5	**500**	**130**	9.7	5.4	27.2	S
Ascension	2	-8.0	-14.4	**500**	**130**	20.2	5.1	26.4	S
Azores	3	38.5	-28.4	1100	124	1.1	15.5	46.0	M
Balleny	4	-66.8	163.3	**500**	**130**	5.7	19.5	51.8	M
Bermuda	5	32.0	-65.0	1100	**168**	18.6	118.3	8.7	S
Bouvet	6	-54.5	3.5	400	**157**	6.8	5.8	28.2	S
Bowie	7	53.5	-135.6	300	**155**	42.2	16.1	47.1	0
Canary	8	28.0	-18.0	1000	164	4.3	153.4	85.2	L
Cape Verde	9	15.0	-24.0	1600	114	7.4	129.8	85.0	L
Caroline	10	5.0	164.0	1600	150	89.3	152.4	85.2	S
Cobb	11	46.0	-130.0	300	**155**	44.7	0.5	6.0	0
Comores	12	-11.8	43.3	**615**	160	16.3	135.2	85.0	M
Crozet	13	-46.3	52.0	500	145	9.0	70.0	81.8	M
Discovery	14	-42.0	0.0	500	**158**	16.4	68.0	81.6	0
Easter	15	-27.1	-109.5	3300	**204**	60.6	6.0	28.7	S
Fernando	16	-4.0	-32.5	500	176	20.0	99.1	84.1	S
Galapagos	17	-0.4	-91.5	1000	130	47.6	12.0	40.5	L
Gough	18	-40.0	10.0	**308**	155	16.3	29.7	63.6	S
Hawaii	19	19.4	-155.3	8700	290	80.5	91.2	83.8	L
Heard Is	20	-53.0	73.0	**1785**	179	8.6	98.0	84.1	M
Iceland	21	65.0	-19.0	1400	186	15.9	8.6	34.4	L
Jan Mayen	22	71.1	-8.2	600	74	2.9	14.6	44.7	M
Juan Fernandez	23	-33.5	-82.0	1600	185	61.8	28.8	62.7	S
Kerguelen	24	-49.6	69.5	500	209	8.9	84.5	83.3	L
Lord Howe	25	-32.0	159.0	900	**165**	63.7	77.2	82.7	S
Louisville	26	-51.0	-138.0	900	200	78.7	42.2	73.6	0
Macdonald	27	-29.0	-140.2	3300	**204**	88.5	40.2	72.4	S
Madeira	28	33.0	-17.0	**677**	161	3.2	133.3	85.0	M
Marion	29	-46.9	37.8	**500**	**130**	8.7	29.5	63.4	M
Marquesas	30	-11.0	-138.0	3300	167	89.0	48.9	76.8	M
Meteor	31	-52.0	1.0	500	**158**	16.3	22.8	55.9	0
New England	32	30.0	-28.0	500	**158**	3.4	87.6	83.5	0
Pitcairn	33	-24.5	-129.0	3300	189	89.8	23.5	56.8	S
Reunion	34	-21.2	55.7	1900	176	16.3	66.7	81.4	M
Samoa	35	-14.5	-168.0	1600	223	88.7	107.0	84.4	L
San Felix	36	-26.3	-80.0	1600	**176**	60.6	38.3	71.1	S
Socorro	37	18.7	-111.0	500	**158**	63.6	1.1	13.3	S
St. Helena	38	-16.0	-6.0	500	164	14.3	38.6	71.3	S
Tahiti	39	-17.9	-148.1	3300	185	89.7	65.8	81.2	M
Tasmanid	40	-39.0	156.0	900	**165**	65.4	53.1	78.3	0
Trindade	41	-20.5	-28.8	500	242	19.8	76.4	82.6	S
Tristan	42	-37.0	-13.0	1700	176	16.2	19.8	52.1	S
Vema	43	-31.5	8.5	400	**157**	16.2	105.6	84.4	0

Supplementary Table 2: *Parameters of global plume melting model. See Methods for information on data sources and on how missing data (bold) was handled. Island categories are S=small (radius less than 10 km), M=medium (radius less than 30 km) and L=large (radius greater than 30 km). "0" indicates that the hotspot is submarine.*

Mid-ocean ridges:

**Baseline global magma flux:**	22.8 km^3 yr^{-1}	
**Baseline global CO_2 flux:**	0.0961 Gt CO_2 yr^{-1}	
**CO_2 concentration mantle source:**	140 ppm_w	

**Δ sea level (m)**	**Duration (kyr)**	**Δ magma flux (km^3 yr^{-1})**	**Δ magma flux (%)**	**Δ CO_2 flux (Gt CO_2 yr^{-1})**	**Δ CO_2 flux (%)**	**Gt CO_2/10 m**	**Total Δ magma (km^3)**	**Total Δ CO_2 (Gt)**
60	5	8.37	36.7	0.0376	39.3	31.4	41850	188
60	15	2.78	12.2	0.0125	13.1	31.4	41700	188
80	10	5.58	24.5	0.0251	26.2	31.4	55800	251
80	15	3.66	16.1	0.0168	17.5	31.5	54900	252
100	10	6.99	30.7	0.0314	32.9	31.4	69900	314
100	15	4.65	20.4	0.0210	21.9	31.5	69750	315

Oceanic mantle plumes:

**Baseline global magma flux:**	2.023	km^3 yr-1
**Baseline global CO_2 flux:**	0.1268	Gt CO_2 yr-1
**CO_2 concentration mantle source:**	950 ppm_w	

**Δ sea level (m)**	**Duration (kyr)**	**Δ magma flux (km^3 yr^{-1})**	**Δ magma flux (%)**	**Δ CO_2 flux (Gt CO_2 yr^{-1})**	**Δ CO_2 flux (%)**	**Gt CO_2/10 m**	**Total Δ magma (km^3)**	**Total Δ CO_2 (Gt)**
60	5	0.69	33.9	0.0423	33.4	35.3	3430	212
60	15	0.24	11.9	0.0172	13.6	43.0	3600	258
80	10	0.53	26.1	0.0328	25.9	41.0	5290	328
80	15	0.35	17.4	0.0233	18.4	43.7	5265	350
100	10	0.63	31.0	0.0383	30.2	38.3	6270	383
100	15	0.41	20.4	0.0264	20.8	39.6	6180	396

Analysed scenarios:

**Scenario**	**Δ sea level (m)**	**Duration (kyr)**	**MOR Δ magma flux (km^3 yr^{-1})**	**MOR Δ CO_2 (Gt CO_2 yr^{-1})**	**Plumes Δ magma flux (km^3 yr^{-1})**	**Plumes Δ CO_2 flux (Gt CO_2 yr^{-1})**	**Total Δ magma (km^3)**	**Total Δ CO_2 (Gt)**
S1	60	5	8.37	0.0376	0.69	0.0423	45280	400
S2	60	15	2.78	0.0125	0.24	0.0172	45300	446
S3	80	15	3.66	0.0168	0.35	0.0233	60165	601
S4	100	10	6.99	0.0314	0.63	0.0383	76170	697

Supplementary Table 3: *Summary of the geodynamic simulations. Scenarios 1-4 have been used to assess the impact of sea level induced volcanic degassing on atmospheric CO_2 levels.*

Variable	Description	Value(s)	Unit
A	Pre-exponential factor in viscosity law	-	1
A_{max}	Maximum viscosity increase during dehydration	5, 10, 50, 100	1
c_p	Specific heat capacity	1100	J kg^{-1} K^{-1}
D_{CO2}	Partition coefficient for CO_2	0.01	1
D_{H2O}	Partition coefficient for H_2O	0.01	1
E_a	Activation energy	400,000	J mol^{-1}
e_z	Unit vector in vertical direction	-	1
F	Depletion (cumulative degree of melting)	-	1
g	Gravitational acceleration	9.81	m s^{-2}
G	Shear modulus	25, 30, 35	GPa
ΔH	(Latent) heat of fusion	660,000	J kg^{-1}
Δh_{SL}	Amplitude of sea level drop	60, 80, 100	m
h_L	Thickness of lithospheric plate at hot spot	50, 70, 90	km
h_{Isl}	Island height above sea floor	7, 10, 13	km
h_w	Water depth	4500	m
k	Thermal conductivity	3	W m^{-1} K^{-1}
p	Pressure	-	Pa
Q_B	Plume buoyancy flux	500, 1000, 4000, 6000, 10000	kg s^{-1}
R	Ideal gas constant	8.314472	J mol^{-1} K^{-1}
ΔS	Entropy of fusion	$\Delta H/T$	J kg^{-1} K^{-1}
t	Time	-	s
Δt_{SL}	Duration of sea level drop	5, 10, 15	kyr
T	Potential temperature	-	ºC
T_M	Reference potential temperature	1335	ºC
T_e	Elastic thickness of lithosphere	15, 25, 35	km
T_{exc}	Plume excess temperature	100, 200, 300	ºC
T^s	Solidus temperature	-	ºC
T_0^s	Solidus temperature at surface (upper mantle/plume)	1081 / 1081	ºC
$\partial T^s/\partial p$	Solidus-pressure gradient (upper mantle/plume)	132 / 112	ºC GPa^{-1}
$\partial T^s/\partial F$	Solidus-depletion gradient (upper mantle/plume)	350 / 250	ºC
V_a	Activation volume	$4\cdot 10^{-6}$	m^3 mol^{-1}
v_i	Velocity component	-	mm yr^{-1}
v_{HS}	Half-spreading rate of mid-ocean ridge	2, …, 100	mm/yr
v_L	Speed of lithospheric plate relative to hot spot	10, 40, 80	mm/yr
x_i	Spatial coordinate	-	m
X^{H2O}	Water content of mantle rock	-	ppm$_w$
X_0^{H2O}	Initial water content of mantle rock	-	ppm$_w$
X_{UM}^{H2O}	Initial water content of upper mantle	50, 100, 200	ppm$_w$
X_P^{H2O}	Initial water content of mantle plumes	400	ppm$_w$
α	Thermal expansion coefficient	$3\cdot 10^{-5}$	ºC^{-1}
β	Depletion-buoyancy parameter	$3\cdot 10^{-2}$	1
η	Dynamic viscosity	-	Pa s
η_0	Reference dynamic viscosity	10^{19}	Pa s
ρ	Density	-	kg m^{-3}
ρ_0	Reference density	3300	kg m^{-3}
ρ_L	Density of oceanic lithosphere	3300	kg m^{-3}
ρ_w	Density of sea water	1030	kg m^{-3}
τ_{ij}	Viscous stress-strain rate tensor	-	Pa
χ^{H2O}	Weight fraction of water in mantle rock	-	1

Supplementary Table 4: List of all symbols and model parameters.

		X_{UM}^{H2O} = 50 ppm$_w$			X_{UM}^{H2O} = 100 ppm$_w$			X_{UM}^{H2O} = 200 ppm$_w$		
		baseline	increase	increase (%)	baseline	increase	increase (%)	baseline	increase	increase (%)
A_{max} = 100	Melt (km^3 yr^{-1})	22.10	2.84	12.9	22.41	2.93	13.1	22.78	3.03	13.3
	CO_2 (Gt yr^{-1})	0.0899	0.0121	13.5	0.0954	0.0134	14.0	0.1027	0.0150	14.6
A_{max} = 50	Melt (km^3 yr^{-1})	22.52	2.70	12.0	**22.80**	**2.78**	**12.2**	23.17	2.85	12.3
	CO_2 (Gt yr^{-1})	0.0907	0.0112	12.3	**0.0961**	**0.0125**	**13.0**	0.1033	0.0141	13.6
A_{max} = 10	Melt (km^3 yr^{-1})	23.12	2.48	10.7	23.41	2.55	10.9	23.78	2.65	11.1
	CO_2 (Gt yr^{-1})	0.0918	0.0101	11.0	0.0970	0.0115	11.9	0.1042	0.0131	12.6
A_{max} = 5	Melt (km^3 yr^{-1})	23.24	2.44	10.5	23.53	2.51	10.7	23.90	2.59	10.8
	CO_2 (Gt yr^{-1})	0.0919	0.0099	10.8	0.0971	0.0114	11.7	0.1043	0.0129	12.4

Supplementary Table 5: Sensitivity tests for the global MOR melting model. The predicted global magma and CO_2 fluxes are shown for different initial water contents X_{UM}^{H2O} of the mantle source (columns) and different factors of viscosity increase A_{max} during melting-induced dehydration (rows). Bold values mark the parameter combination used for the model runs presented in the main text.

Reference plume model presented in main text

	baseline	increase	increase (%)
Melt production ($km^3\ yr^{-1}$)	2.023	0.240	11.9
CO_2 release ($Gt\ yr^{-1}$)	0.1268	0.0172	13.6

Predicted magma and CO_2 fluxes when varying one parameter

Model parameter		Parameter variation: -10%			+10%		
		baseline	increase	increase (%)	baseline	increase	increase (%)
Buoyancy flux	Melt ($km^3\ yr^{-1}$)	1.941	0.237	12.2	2.117	0.243	11.5
	CO_2 ($Gt\ yr^{-1}$)	0.1216	0.0170	14.0	0.1328	0.0174	13.1
Excess temperature	Melt ($km^3\ yr^{-1}$)	1.679	0.227	13.5	2.355	0.252	10.7
	CO_2 ($Gt\ yr^{-1}$)	0.1173	0.0176	15.0	0.1347	0.0169	12.5
Plate speed at hotspot	Melt ($km^3\ yr^{-1}$)	1.998	0.245	12.3	2.035	0.236	11.6
	CO_2 ($Gt\ yr^{-1}$)	0.1253	0.0175	14.0	0.1279	0.0169	13.2
Lithosphere thickness	Melt ($km^3\ yr^{-1}$)	2.329	0.289	12.4	1.798	0.204	11.3
	CO_2 ($Gt\ yr^{-1}$)	0.1349	0.0191	14.2	0.1211	0.0156	12.9

Change in predicted fluxes relative to reference model

Model parameter		Parameter variation: -10%			+10%		
		baseline	increase	increase (%)	baseline	increase	increase (%)
Buoyancy flux	Change in melt (%)	-4.1	-1.3	2.9	4.6	1.3	-3.2
	Change in CO_2 (%	-4.1	-1.2	3.1	4.7	1.2	-3.4
Excess temperature	Change in melt (%)	-17.0	-5.4	14.0	16.4	5.0	-9.8
	Change in CO_2 (%	-7.5	2.3	10.6	6.2	-1.7	-7.5
Plate speed at hotspot	Change in melt (%)	-1.2	2.1	3.4	0.6	-1.7	-2.2
	Change in CO_2 (%	-1.2	1.7	3.0	0.9	-1.7	-2.6
Lithosphere thickness	Change in melt (%)	15.1	20.4	4.6	-11.1	-15.0	-4.4
	Change in CO_2 (%	6.4	11.0	4.4	-4.5	-9.3	-5.0

Supplementary Table 6: Sensitivity tests for the global plume melting model. The predicted magma and CO_2 fluxes are most sensitive to changes in the plume excess temperature and the lithosphere thickness at the hotspot location. The model results depend less on the plume buoyancy fluxes and are rather insensitive to the plate speed at the hotspot location. Note that these are the integrated global values and that individual hotspots might show stronger variations.

Supplementary References

1 Jouzel, J. *et al.* Orbital and millennial Antarctic climate variability over the past 800,000 years. *Science* **317**, 793-796, doi:10.1126/science.1141038 (2007).

2 Bereiter, B. *et al.* Revision of the EPICA Dome C CO_2 record from 800 to 600kyr before present. *Geophysical Research Letters* **42**, 542-549, doi:10.1002/2014gl061957 (2015).

3 Lambert, F. *et al.* Dust-climate couplings over the past 800,000 years from the EPICA Dome C ice core. *Nature* **452**, 616-619, doi:10.1038/nature06763 (2008).

4 Grant, K. M. *et al.* Sea-level variability over five glacial cycles. *Nature Communications* **5**, doi:10.1038/ncomms6076 (2014).

5 Bintanja, R., van de Wal, R. S. W. & Oerlemans, J. Modelled atmospheric temperatures and global sea levels over the past million years. *Nature* **437**, 125-128, doi:10.1038/nature03975 (2005).

6 Veres, D. *et al.* The Antarctic ice core chronology (AICC2012): an optimized multi-parameter and multi-site dating approach for the last 120 thousand years. *Climate of the Past* **9**, 1733-1748, doi:10.5194/cp-9-1733-2013 (2013).

7 Bazin, L. *et al.* An optimized multi-proxy, multi-site Antarctic ice and gas orbital chronology (AICC2012): 120-800 ka. *Climate of the Past* **9**, 1715-1731, doi:10.5194/cp-9-1715-2013 (2013).

8 Laskar, J. *et al.* A long-term numerical solution for the insolation quantities of the Earth. *Astron. Astrophys.* **428**, 261-285, doi:10.1051/0004-6361:20041335 (2004).

9 Köhler, P., Fischer, H. & Schmitt, J. Atmospheric $\delta^{13}CO_2$ and its relation to pCO_2 and deep ocean δC^{13} during the late Pleistocene. *Paleoceanography* **25**, doi:10.1029/2008pa001703 (2010).

10 Martinez-Garcia, A. *et al.* Iron Fertilization of the Subantarctic Ocean During the Last Ice Age. *Science* **343**, 1347-1350, doi:10.1126/science.1246848 (2014).

11 Huybers, P. & Langmuir, C. Feedback between deglaciation, volcanism, and atmospheric CO_2. *Earth and Planetary Science Letters* **286**, 479-491, doi:10.1016/j.epsl.2009.07.014 (2009).

12 Medina-Elizalde, M. A global compilation of coral sea-level benchmarks: Implications and new challenges. *Earth and Planetary Science Letters* **362**, 310-318, doi:10.1016/j.epsl.2012.12.001 (2013).

13 Grant, K. M. *et al.* Rapid coupling between ice volume and polar temperature over the past 150,000 years. *Nature* **491**, 744-747, doi:10.1038/nature11593 (2012).
